# Efficacy and safety of combined Chinese and Western medicine in the treatment of knee osteoarthritis: a prospective, multicenter cohort study

**DOI:** 10.3389/fphar.2023.1176980

**Published:** 2023-08-28

**Authors:** Qian-Yun Ye, Qing Lin, Xue-Ling Hu, Yu-Mei Yang, Bao-Lin Zheng, Ting Li, Wen-Qiang Zhong, Hao-Yu Wang, Zhi-Fen Zhang, Bing-Jie Luo, Ya-Wen Xiao, Ai-Ling Wu, Yan Li, Zhuo-Ling Zou, Ling-Yu Li, Xiao-Yun Li, Pan-Pan Wang, Li Yang, Xiao-Feng Zhu, Li Han, Rong-Hua Zhang

**Affiliations:** ^1^ College of Traditional Chinese Medicine, Jinan University, Guangzhou, China; ^2^ First Affiliated Hospital of Jinan University, Guangzhou, China; ^3^ College of Pharmacy, Jinan University, Guangzhou, China; ^4^ Department of Nephropathy and Rheumatology, Foshan Hospital of TCM, Foshan, China; ^5^ Guangdong Provincial Key Laboratory of Traditional Chinese Medicine Informatization, Jinan University, Guangzhou, China; ^6^ Cancer Research Institution, Jinan University, Guangzhou, China

**Keywords:** knee osteoarthritis, combined Chinese and Western medicine, prospective cohort, efficacy, real-world study

## Abstract

**Purpose:** To conduct a real-world evaluation of the efficacy and safety of combined Chinese and Western medicine in treating knee osteoarthritis (KOA).

**Methods:** A multicenter, prospective cohort study design was employed, enrolling 450 KOA patients (Kellgren-Lawrence score of 3 or less). The patients were divided into a Western medicine treatment group (WM group) and a combined Western and traditional Chinese medicine treatment group (WM-CM group). A 6-week treatment plan was administered, and follow-up visits occurred at 2 weeks, 4 weeks, and 6 weeks after initiating treatment. The primary outcome indicator was the total Western Ontario and McMaster Universities Arthritis Index (WOMAC) score after 6 weeks of treatment. Secondary outcome indicators included WOMAC subscales for pain, stiffness, and joint function, visual analogue scale (VAS) score, physical component summary (PCS), mental component summary (MCS), and clinical effectiveness. The incidence of drug-related adverse events was used as a safety evaluation indicator.

**Results:** A total of 419 patients were included in the final analysis: 98 in the WM group and 321 in the WM-CM group. The baseline characteristics of the two groups were comparable, except for the incidence of stiffness symptoms and stiffness scores. After 6 weeks of treatment, the WM-CM group exhibited superior results to the WM group in improving the total WOMAC score (24.71 ± 1.38 vs. 16.36 ± 0.62, *p* < 0.001). The WM-CM group also outperformed the WM group in WOMAC pain and joint function scores, VAS score, PCS score, MCS score, and clinical effectiveness (*p* < 0.05), which was consistent with the findings of the main evaluation index. Subgroup analysis indicated that the combined Chinese and Western medicine treatment showed more pronounced benefits in patients under 65 years of age and in those with a Kellgren-Lawrence (K-L) classification of 0-I. Throughout the study, no adverse effects were observed in either group.

**Conclusion:** The combination of Chinese and Western medicine demonstrated superiority over Western medicine alone in relieving knee pain symptoms, improving knee function, and enhancing the quality of life for KOA patients with a K-L score of 3 or less. Moreover, the treatment exhibited a good safety profile.

**Clinical Trial Registration:** (https://www.chictr.org.cn/), identifier (ChiCTR1900027175).

## 1 Introduction

Knee osteoarthritis (KOA) is one of the most common musculoskeletal disorders and a major cause of disability in elderly individuals (Collaborators, 2018; Jang S., 2021; Lundberg M., 2022). With the accelerated aging process, the growth of the obese population and the increasing life expectancy, the prevention and treatment of KOA are facing a serious challenge (Mahmoudian A., 2021). According to statistics, KOA has affected more than 250 million people worldwide (Vos T., 2012), and in China, the prevalence of symptomatic KOA in people over 65 years of age is 60%, and the detection rate of radiological KOA is as high as 80% (Huang D., 2018). Moreover, the number of patients with KOA will continue to increase in the future, and it is estimated that it will be close to 400 million by 2030 (Pereira D., 2011; Zhang Z., 2020).

KOA is characterized by a long, irreversible and incurable course, causing pain, reduced mobility and even disability, which places a great burden on patients’ physical and mental health and seriously interferes with their quality of life (Wang X., 2016; Feng X.Q., 2022). In addition, the high medical costs and corresponding indirect costs of KOA not only increase the economic burden of individuals and families but also have a negative impact on the national healthcare system and increase socioeconomic costs (Tang X., 2016).

Conservative therapy is now becoming increasingly important in the long-term management of KOA as the first line of treatment to slow disease progression and avoid or delay knee replacement surgery (Lim W.B., 2022). Western conservative treatment is mainly based on pain control and cartilage nutrition protocols, with the point of action localized to the joint. In fact, KOA involves multiple lesions and complex pathological changes, which are the result of multiple pathogenic factors intertwined and acting over a long period of time, and the efficacy of localized, single western medicine treatment is limited (Li R., 2013).

Traditional Chinese medicine (TCM) has a history of thousands of years in treating KOA, with a wide variety of therapeutic approaches that can play a role in holistic conditioning, and it is widely used in China and other Asian countries. Although there is preliminary evidence that TCM has the advantage of significant efficacy and low adverse effects and can improve the clinical efficacy of KOA in conjunction with Western medicine, overall, the evidence from high-quality clinical studies is still very limited (Chen B., 2016; Zhang J.H., 2019; Deng Y.L., 2021). Therefore, based on the concept of addressing the characteristics of individualized complex interventions and holistic efficacy evaluation of TCM, we conducted a prospective, multicenter cohort study following the requirements of modern clinical epidemiology and evidence-based medicine to further evaluate the clinical efficacy and safety of combined Chinese and Western medicine in the treatment of KOA from a macroscopic perspective to support its early intervention in KOA.

## 2 Methods and materials

### 2.1 Study design

This study was conducted as a prospective, multicenter cohort study from January 2021 to October 2022 in three medical institutions: the First Affiliated Hospital of Jinan University, the Third Affiliated Hospital of Guangzhou University of Traditional Chinese Medicine, and Foshan Hospital of Traditional Chinese Medicine. It was approved by the Ethics Committee of the First Hospital of Jinan University (KY-2019-036), and registration was completed at the China Clinical Trials Registry (ChiCTR1900027175). All subjects provided written informed consent.

### 2.2 Inclusion and exclusion criteria

The inclusion criteria for the study population were as follows: 1) meeting the diagnostic criteria for KOA and having a Kellgren-Lawrence (K-L) radiological diagnosis grade of III or less, based on the Guidelines for the Treatment of Knee Osteoarthritis with Integrative Medicine (Committee, 2018); 2) being aged 30 years or older; 3) not having plans for surgery in the near future and requiring conservative treatment; and 4) demonstrating good compliance and the ability to cooperate with the completion of clinical visits.

Exclusion criteria were as follows: 1) having a history of knee trauma or surgery in the last 6 months, along with knee fracture, dislocation, or septic knee arthritis; 2) receiving arthroscopic treatment or intra-articular injection in the last 3 months; 3) receiving hormone therapy in the last 1 month; 4) having undergone knee replacement; 5) having comorbidities such as tumor, tuberculosis, hemophilic arthritis, rheumatoid arthritis, systemic lupus erythematosus, or ankylosing spondylitis; 6) having serious gastrointestinal diseases, severe psychiatric disorders, significant infectious diseases, or severe pathologies affecting vital organs such as the heart, liver, kidneys, or others; 7) having a history of severe allergy to TCM; 8) having local skin ulcers or eczema; and 9) being deemed unsuitable for inclusion in the study by the investigator.

### 2.3 Therapeutic strategy

The patients were divided into a Western medicine treatment group (WM group) and a combined Western and traditional Chinese medicine treatment group (WM-CM group) according to the actual treatment protocol used in the clinic. All subjects received basic treatment, including health education and exercise instruction. In the WM group, NSAIDs, sodium glutamate and glucosamine were used as the main treatment drugs according to the relevant guidelines (Bannuru R.R., 2019; Association, 2021; Zhang Z., 2021), while the WM-CM group used a combination of Western and traditional Chinese medicine according to the comprehensive treatment plan we developed in the early stage. All patients included in the study received a 6-week treatment, and the medication was administered following the recommended conventional dosage as per the instructions. The types of medications used during treatment were shown in Table 1. TCM therapy mainly includes commercially available Chinese patent medicines and hospital preparations that have been used for many years. We provided detailed information on the most frequently used oral and topical traditional Chinese medicines in Table 2, including their composition, proportions, dosage, usage, manufacturing processes, and quality control aspects. The detailed information for the other traditional Chinese medicines can be found in Supplementary Tables S1, S2 of the supplementary materials.

**TABLE 1:** The frequency of drug usage during treatment.

Medication type	Overall no. (%)	WM group no. (%)	WM-CM group no. (%)
Sodium Citrate	377 (90.0)	93 (94.9)	284 (88.5)
Glucosamine	119 (28.4)	43 (43.9)	76 (23.7)
NSAIDs			
Overall use of NSAIDs	230 (54.9)	75 (76.5)	155 (48.3)
Aceclofenac	102 (24.3)	7 (7.1)	95 (29.6)
Diclofenac Sodium	56 (13.4)	41 (41.8)	15 (4.7)
Etoricoxib	26 (6.2)	7 (7.1)	19 (5.9)
Celecoxib	22 (5.3)	10 (10.2)	12 (3.7)
Flurbiprofen Gel Patch	21 (5.0)	19 (19.4)	2 (0.6)
Loxoprofen Sodium	16 (3.8)	7 (7.1)	9 (2.8)
Meloxicam	14 (3.3)	3 (3.1)	11 (3.4)
Others	5 (1.2)	5 (5.1)	0 (0.0)
Oral Chinese medicine			
Duhuo Jisheng Mixture or Decoction			61 (19.0)
Yuxuebi Tablet			49 (15.3)
Gulong Capsule			29 (9.0)
Wangbi Tablet			23 (7.2)
Yougui Capsule			19 (5.9)
Zhongtongan Capsule			17 (5.3)
Zhuifeng Tougu Capsule			13 (4.0)
Hugu Capsule			13 (4.0)
Tenghuang Jiangu Tablet			12 (3.7)
Dahuoluo Capsule			10 (3.1)
Xianling Gubao Capsule			7 (2.2)
Others			19 (5.9)
External Chinese medicine			
Jianbu Xiaozhong Zhitong Oil			110 (34.3)
Warming Jingjintong Plaster			100 (31.2)
Cooling Blood Swelling Ointment			30 (9.3)
Cooling Jingjintong Plaster			18 (5.6)
Wentong Ointment			14 (4.4)
Li Guanghai Dieda Qufeng Ointment			13 (4.0)
Tianbai Golden Plaster			13 (4.0)
Antai Gel Ointment			12 (3.7)
Huoxue Powder			11 (3.4)
Daiwenjiu Ointment			11 (3.4)
Others			35 (10.9)

**TABLE 2:** Details of the most common oral and topical traditional Chinese medicines in this study.

Medication type	Ingredients	Percentage (%)	Processing	Approval number	Executive standard	Usage and dosage
Oral Chinese medicine						
Duhuo Jisheng Mixture^*^	Angelica Biserrata (R.H.Shan and C.Q.Yuan) C.Q.Yuan and R.H.Shan[Apiaceae; Angelicae Pubescentis Radix (Du-Huo)]	9.7	Gentiana Macrophylla Pall.[Gentianaceae; Gentianae Macrophyllae Radix (Qin-Jiao) ], Paeonia Lactiflora Pall.[Paeoniaceae; Paeoniae Radix Alba (Bai-Shao)] and Eucommia Ulmoides Oliv.[Eucommiaceae; Eucommiae Cortex (Yan-Du-Zhong)] are extracted with 70% ethanol; volatile oils are extracted from Angelica Biserrata (R.H.Shan and C.Q.Yuan) C.Q.Yuan and R.H.Shan[Apiaceae; Angelicae Pubescentis Radix (Du-Huo)], Asarum Heterotropoides F.Schmidt[Aristolochiaceae; Asari Radix Et Rhizome (Xi-Xin)], Neolitsea Cassia (L.) Kosterm.[Lauraceae; Cinnamomi Ramulus (Gui-Zhi)], Saposhnikovia Divaricata (Turcz. Ex Ledeb.) Schischk.[ Apiaceae; Saposhnikoviae Radix (Fang-Feng) ], Angelica sinensis (Oliv.) Diels[Apiaceae; Angelicae Sinensis Radix (Dang-Gui)] and Conioselinum Anthriscoides “Chuanxiong”[Apiaceae; Chuanxiong Rhizome (Chuan-Xiong)]; all the medicines are decocted with water and mixed with ethanol extracts and volatile oils.	Z10983003	Chinese Pharmacopoeia 2015 Edition Part One	20 mL each time, 3 times a day
	Taxillus Chinensis (Dc.) Danser[Loranthaceae; Taxilli Herba (Sang-Ji-Sheng)]	6.4				
	Gentiana Macrophylla Pall.[Gentianaceae; Gentianae Macrophyllae Radix (Qin-Jiao) ]	6.4				
	Saposhnikovia Divaricata (Turcz. Ex Ledeb.) Schischk.[Apiaceae; Saposhnikoviae Radix (Fang-Feng) ]	6.4				
	Asarum Heterotropoides F.Schmidt[Aristolochiaceae; Asari Radix Et Rhizome (Xi-Xin)]	6.4				
	Angelica sinensis (Oliv.) Diels[Apiaceae; Angelicae Sinensis Radix (Dang-Gui)]	6.4				
	Paeonia Lactiflora Pall.[Paeoniaceae; Paeoniae Radix Alba (Bai-Shao)]	6.4				
	Conioselinum Anthriscoides “Chuanxiong”[Apiaceae; Chuanxiong Rhizome (Chuan-Xiong)]	6.4				
	Rehmannia Glutinosa (Gaertn.) DC.[Orobanchaceae; Rehmanniae Radix Praeparata (Shu-Di-Huang)]	6.4				
	Eucommia Ulmoides Oliv.[Eucommiaceae; Eucommiae Cortex (Yan-Du-Zhong)]	6.4				
	Cyathula Officinalis K.C.Kuan[Amaranthaceae; Cyathulae Radix (Chuan-Niu-Xi) ]	6.4				
	Codonopsis Pilosula (Franch.) Nannf.[Campanulaceae; Codonopsis Radix (Dang-Shen)]	6.4				
	Carapichea Ipecacuanha (Brot.) L.Andersson[Polyporaceae; Poria (Fu-Ling)]	6.4				
	Glycyrrhiza Glabra L.[Fabaceae; Glycyrrhizae Radix Et Rhizoma (Gan-Cao)]	6.4				
	Neolitsea Cassia (L.) Kosterm.[Lauraceae; Cinnamomi Ramulus (Gui-Zhi)]	6.4				
Yuxuebi Tablet^*^	Boswellia Sacra Flück. [Burseraceae; Olibanum (Ru-Xiang)]	4.5	Take Cyathula Officinalis K.C.Kuan [Amaranthaceae; Cyathulae Radix (Chuan-Niu-Xi) ] and half the amount of Salvia Miltiorrhiza Bunge[Labiatae; Salviae Miltiorrhizae Radix Et Rhizoma (Dan-Shen)] and Astragalus Mongholicus Bunge[Fabaceae; Astragali Radix(Zhi-Huang-Qi)], and grind them into fine powder. Decoct the remaining drugs and concentrate under reduced pressure to make a clear paste. The paste is mixed with crude drug powder to make granules, and then compressed into tablets.	Z20050762	State Food and Drug Administration Drug Standards YBZ28152005-2009Z	5 tablets at a time, 3 times a day
	Clematis Chinensis Osbeck [Ranunculaceae; Clematidis Radix Et Rhizoma (Wei-Ling-Xian)]	11.2				
	Carthamus Tinctorius L. [Asteraceae; Carthami Flos (Hong-Hua)]	7.5				
	Salvia Miltiorrhiza Bunge[Labiatae; Salviae Miltiorrhizae Radix Et Rhizoma (Dan-Shen)]	14.9				
	Commiphora Myrrha (T.Nees) Engl.[Burseraceae; Myrrha(Zhi-Mo-Yao)]	4.5				
	Cyathula Officinalis K.C.Kuan[Amaranthaceae; Cyathulae Radix (Chuan-Niu-Xi) ]	11.2				
	Conioselinum Anthriscoides ‘Chuanxiong'[Apiaceae; Chuanxiong Rhizome (Chuan-Xiong)]	11.2				
	Angelica sinensis (Oliv.) Diels[Apiaceae; Angelicae Sinensis Radix (Dang-Gui)]	7.5				
	Curcuma Longa L.[Zingiberaceae; Curcumae Longae Rhizoma(Jiang-Huang)]	7.5				
	Cyperus Rotundus L.[Cyperaceae; Cyperi Rhizoma(Zhi-Xiang-Fu)]	9.0				
	Astragalus Mongholicus Bunge[Fabaceae; Astragali Radix(Zhi-Huang-Qi)]	11.2				
**External Chinese medicine**						
Jianbu Xiaozhong Zhitong Oil^$^	Styrax benzoin Dryand.[Styracaceae; Benzoinum(An-Xi-Xiang)]		Styrax benzoin Dryand.[Styracaceae; Benzoinum(An-Xi-Xiang)] benzoin with ethanol for 7 days, and keep the filtrate. Crush Capsicum annuum L.[Solanaceae; Capsici Fructus(La-Jiao)] into fine powder, add Turpentine Oil (Song-Jie You) and soak for 7 days, and keep the filtrate. Mix the Above-mentioned filtrate with L-Menthol (Bo-He-Nao), Clove Oil (Ding-Xiang You), Borneolum Syntheticum (Bing-Pian) and Eucalyptus Oil (An You).	ZB20150003 (Guangdong batch number)	Registration Standards for Preparations in Medical Institutions	3 to 4 times a day
	Capsicum annuum L.[Solanaceae; Capsici Fructus(La-Jiao)]					
	L-Menthol (Bo-He-Nao)					
	Clove Oil (Ding-Xiang You)					
	Borneolum Syntheticum (Bing-Pian)					
	Eucalyptus Oil (An You)					
	Turpentine Oil (Song-Jie You)					
Warming Jingjintong Plaster^$^	Cullen corylifolium (L.) Medik.[Fabaceae; Psoraleae Fructus(Bu-Gu-Zhi)]	7.5	Except for Camphor(Racemic) (Zhang-Nao(He-Cheng)), Borneolum Syntheticum (Bing-Pian), L-Menthol (Bo-He-Nao), and Turpentine Oil (Song-Jie You), the rest drugs are crushed into coarse powder, and extracted with ethanol to make a clear paste. Add carbomer to the clear paste and soak for 24 h, then add Camphor(Racemic) (Zhang-Nao(He-Cheng)), Borneolum Syntheticum (Bing-Pian), L-Menthol (Bo-He-Nao), Turpentine Oil (Song-Jie You), and mix well.	ZB20150006 (Guangdong batch number)	Registration Standards for Preparations in Medical Institutions	3 to 4 times a day
	Astragalus Mongholicus Bunge[Leguminosae; Astragali Radix(Huang-Qi)]	7.5				
	Phellodendron amurense Rupr.[Rutaceae; Phellodendri Amurensis Cortex(Guan-Huang-Bo)	6.0				
	Achyranthes bidentata Blume[Amaranthaceae; Achyranthis Bidentatae Radix(Niu-Xi)]	6.0				
	Dipsacus asper Wall. ex DC.[Dipsacaceae; Dipsaci Radix(Xu-Duan)]	6.0				
	Eucommia Ulmoides Oliv.[Eucommiaceae; Eucommiae Cortex (Du-Zhong)]	4.5				
	Drynaria roosii Nakaike[Polypodiaceae; Drynariae Rhizoma(Gu-Sui-Bu)]	4.5				
	Chaenomeles speciosa (Sweet) Nakai[Rosaceae; Chaenomelis Fructus(Mu-Gua)]	6.0				
	Spatholobus suberectus Dunn[Fabaceae; Spatholobi Caulis(Ji-Xue-Teng)]	4.5				
	Zanthoxylum nitidum (Roxb.) DC.[Rutaceae; Zanthoxyli Radix(Liang-Mian-Zhen)]	4.5				
	Vincetoxicum mukdenense Kitag.[Apocynaceae; Cynanchi Paniculati Radix Et Rhizoma(Xu-Chang-Qing)]	3.0				
	Rheum officinale Baill.[Polygonaceae; Rhei Radix Et Rhizoma(Da-Huang)]	3.0				
	Carthamus Tinctorius L.[Asteraceae; Carthami Flos (Hong-Hua)]	1.5				
	Zingiber officinale Roscoe[Zingiberaceae; Zingiberis Rhizoma(Gan-Jiang)]	1.5				
	Capsicum annuum L.[Solanaceae; Capsici Fructus(La-Jiao)]	1.5				
	Camphor(Racemic) (Zhang-Nao(He-Cheng))	11.2				
	Borneolum Syntheticum (Bing-Pian)	7.5				
	L-Menthol (Bo-He-Nao)	4.1				
	Turpentine Oil (Song-Jie You)	9.7				

Note: ^*^Drugs’ information comes from “China Pharmacopoeia 2020 edition”; ^$^Drugs’ information comes from “Guangdong Province Medical Institution Preparation Standard”. Referring to the publication method of “Chinese Pharmacopoeia”, we briefly describe the prescription and preparation method if it involves confidential technology.

### 2.4 Observation indicators

Patients were evaluated before treatment and at 2, 4, and 6 weeks of treatment.

The primary outcome measure was the total Western Ontario and McMaster Universities Arthritis Index (WOMAC) score (ranging from 0 to 96, with higher scores indicating more severe symptoms). The primary end point is the WOMAC total score after 6 weeks of treatment.

Secondary efficacy indicators included the following:

1) WOMAC subscale scores, including WOMAC pain score (ranging from 0 to 20, with higher scores indicating more severe pain), WOMAC stiffness score (ranging from 0 to 8, with higher scores indicating more severe knee stiffness), and WOMAC joint function score (ranging from 0 to 68, with higher scores indicating poorer knee function);
2) The visual analogue scale (VAS) score (ranging from 0 to 10, with higher scores indicating more severe pain);
3) 36-item short form (SF-36) scores, divided into physical component summary (PCS) and mental component summary (MCS); the former includes four dimensions of physical functioning (PF), role-physical (RP), bodily pain (BP), and general health (GH), and the latter includes four dimensions of vitality (VT), social functioning (SF), role-emotional (RE), and mental health (MH), ranging from 0 to 100, with higher scores indicating better status on that dimension; and
4) Clinical effectiveness. As described in the literature (Angst F., 2001; Nishida Y., 2021), an improvement in the WOMAC total score by more than 12% from the baseline value was defined as effective, and an improvement in the WOMAC total score by more than 50% from the baseline value was defined as significant.

An adverse event was defined as any undesirable medical occurrence associated with the treatment protocol, leading to a persistence or worsening of the patient’s symptoms that required additional interventions (Deyle G.D., 2016). For this study, patients were asked to report at each follow-up visit any adverse outcomes, including complications, signs or symptoms, that they perceived to be related to their treatment. The incidence of adverse events was considered an indicator for safety evaluation.

### 2.5 Sample size

G*Power 3.1.2 software was used to calculate the sample size for this study. Based on the relevant literature (Cao Y., 2005; Deyle G.D., 2020) and the previous study of our group, assuming that the mean WOMAC values of the WM group and the WM-CM group were equal at baseline and that a 12% difference between the two groups was maintained after each treatment, setting a standard deviation of 7 and a mean correlation of 0.681 between repeated measures, and considering a 10% missed visit rate, then with a sample size of 92 cases per group, there is a 90% test efficacy to find the difference between groups. Therefore, we set the minimum sample size at 92 cases and adjusted the sample size appropriately according to the actual situation.

### 2.6 Statistical analysis

SPSS 27.0 software was used for statistical analysis. Data analysis is based on all populations completing the study unless otherwise noted. Hypothesis testing was uniformly performed using a two-sided test, with *p* < 0.05 indicating a statistically significant result. Baseline information was described as the mean ± standard deviation or frequency and composition ratio. Two groups were compared using independent samples t tests or Mann‒Whitney U rank sum tests for measurement data and chi-square tests or Fisher’s exact tests for count data. Repeated measures data were compared between groups using generalized linear mixed models (GLMM). The model included treatment, time, and the interaction of treatment with time as fixed effects and patient-specific random intercepts.

## 3 Results

### 3.1 Population characteristics

A total of 450 patients were enrolled in this study, and 419 subjects completed the entire study process, eventually forming the analysis cohort, consisting of 98 patients in the WM group and 321 patients in the WM-CM group. Among them, there were 100 patients from Foshan Hospital of Traditional Chinese Medicine, including 14 in the WM group and 86 in the WM-CM group; 106 patients from the First Affiliated Hospital of Jinan University, including 70 in the WM group and 36 in the WM-CM group; and 213 patients from the Third Affiliated Hospital of Guangzhou University of Traditional Chinese Medicine, including 14 in the WM group and 199 in the WM-CM group.

**FIGURE 1:**
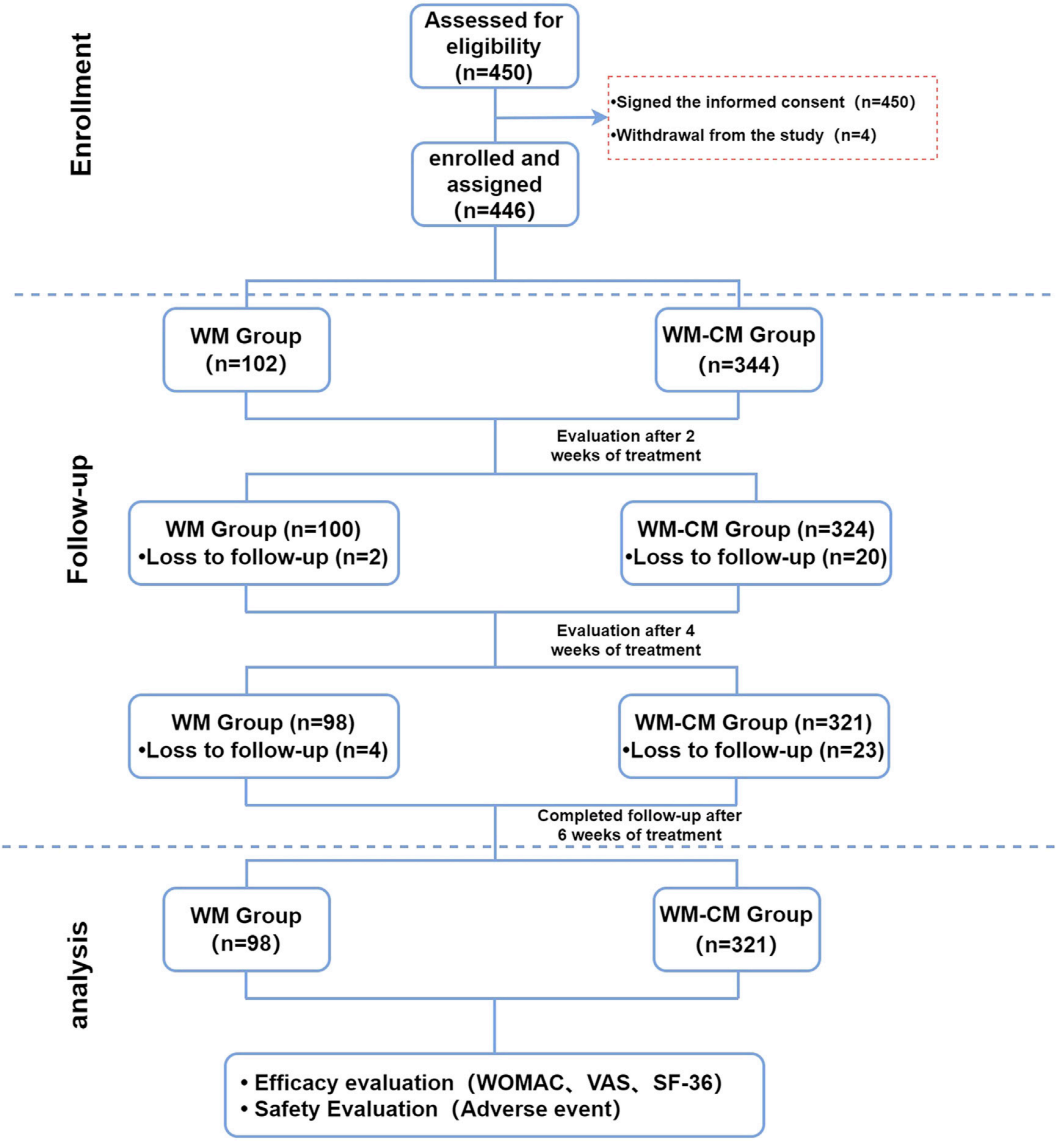
Flow chart of the study.

The patient follow-up process and lost visits are presented in Figure 1. Detailed baseline characteristics of the patients are summarized in Table 3. The prevalence of knee stiffness symptoms was higher in the WM-CM group (*p* = 0.003), while the WOMAC stiffness score was higher in the WM group (*p* = 0.019); otherwise, the rest of the baseline characteristics were comparable.

**TABLE 3:** Baseline characteristics of all the subjects.

Characteristics	WM group (n = 98)	WM-CM group (n = 321)	*p*
Age, years	61.37 ± 8.09	60.98 ± 7.70	0.831
BMI, $kg∙m^{-2}$	24.13 ± 3.28	23.61 ± 2.99^*^	0.138
Sex, no. (%), male/female	23 (23.5)/75 (76.5)	70 (21.8)/251 (78.2)	0.729
Disease duration, no. (%)			
<1 month	5 (5.1)	8 (2.5)	0.064
1–3 months	6 (6.1)	7 (2.2)	
4–6 months	5 (5.1)	10 (3.1)	
>6 months	82 (83.7)	296 (92.2)	
K-L grade, no. (%)			
0	4 (4.1)	38 (6.9)	0.162
I	74 (75.5)	186 (63.2)	
II	16 (16.3)	80 (24.6)	
III	4 (4.1)	17 (5.3)	
Main symptoms of knee, no. (%)			
Pain	97 (99.0)	318 (99.1)	1.000
Restricted movement	66 (67.3)	247 (76.9)	0.056
Stiffness	32 (32.7)	159 (49.5)	**0.003**
Swelling	38 (38.8)	138 (43.0)	0.459
Crepitus	25 (25.5)	112 (34.9)	0.083
Soreness and weakness	29 (29.6)	124 (38.6)	0.104
Baseline measure			
WOMAC total score	37.62 ± 14.74	36.23 ± 12.71	0.461
WOMAC pain score	8.62 ± 4.12	8.35 ± 3.26	0.705
WOMAC stiffness score	2.50 ± 1.61	2.05 ± 1.69	**0.019**
WOMAC physical function score	26.50 ± 10.59	25.83 ± 9.00	0.536
VAS score	5.07 ± 1.72	5.23 ± 1.46	0.352
PCS score	44.51 ± 18.48	41.67 ± 16.53	0.197
MCS score	68.28 ± 19.98	66.10 ± 20.54	0.427

Note: Continuous variables are expressed as the mean ± standard deviation. Bolded values represent *p* <0.05 for comparison between the two groups. ^*^ Values were obtained based on data from 319 patients.

### 3.2 The primary outcome measure

**FIGURE 2:**
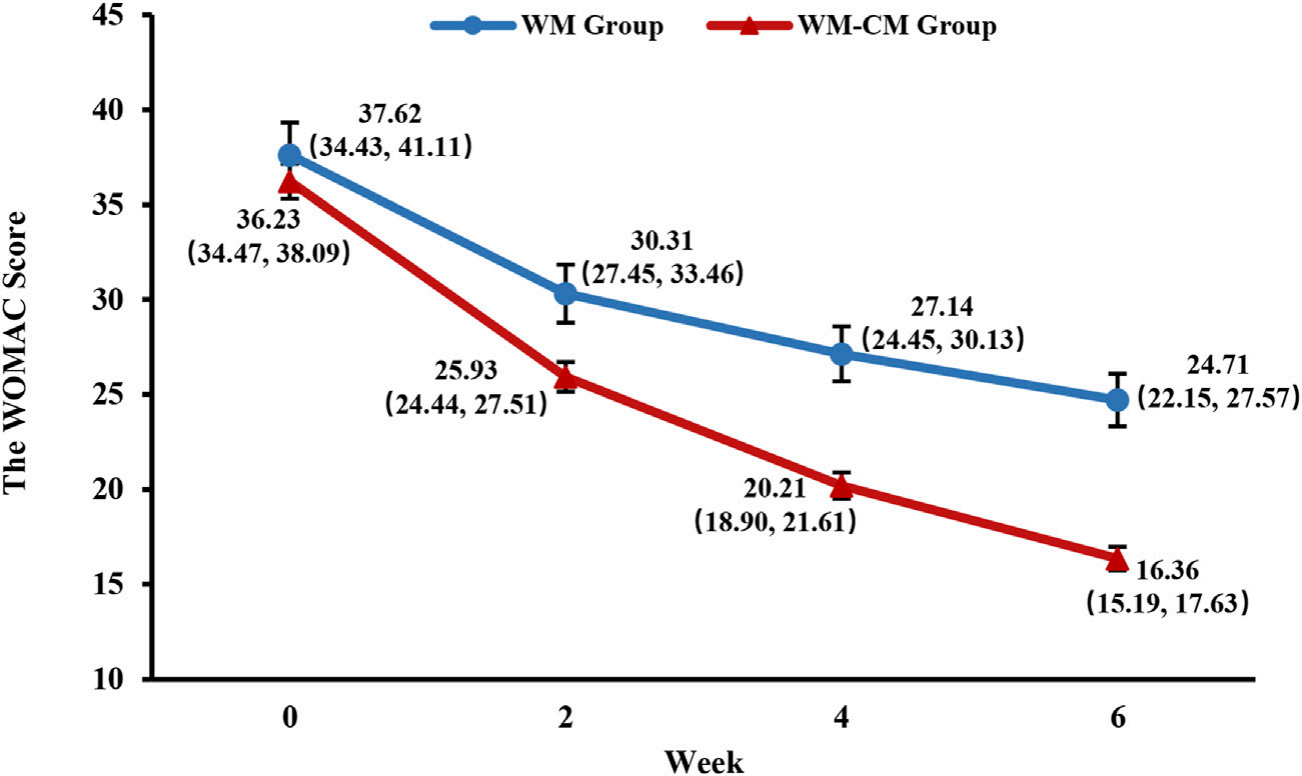
Overall trend of the WOMAC total scores. Note: The values shown are least-squares mean calculated based on generalized linear mixed model, with 95% confidence intervals (indicated by error bars) in parentheses.

The mean total WOMAC values at baseline were 37.62 ± 1.702 in the WM group and 36.23 ± 0.923 in the WM-CM group, and the differences were not statistically significant. After 6 weeks of treatment, the mean WOMAC total score decreased to 24.71 ± 1.38 in the WM group and 16.36 ± 0.62 in the WM-CM group. The mean total WOMAC value was significantly lower in the WM-CM group than in the WM group (*p* < 0.001) (Table 4). Indeed, from the second week of treatment, there was a significant difference in the total WOMAC values of the two groups, and this trend persisted until the sixth week (Figure 2; Supplementary Table S3).

**TABLE 4:** Primary and Secondary outcomes at 6 Weeks.

Outcome	WM group	WM-CM group	*p*
WOMAC total score	24.71 ± 1.38	16.36 ± 0.62	<0.001
WOMAC pain score	5.39 ± 0.342	4.1 ± 0.165	0.001
WOMAC physical function score	17.7 ± 0.986	11.45 ± 0.438	<0.001
VAS score	2.97 ± 0.149	2.24 ± 0.072	<0.001
PCS score	49.09 ± 1.939	59.75 ± 1.304	<0.001
MCS score	71.83 ± 2.131	78.36 ± 1.284	0.009

Note: All 419 patients were included in the analysis.

The least-squares mean ± SD, was calculated based on generalized linear mixed model, and the *p* values were adjusted with the use of Bonferroni correction for multiple comparisons.

### 3.3 The secondary outcome measure

#### 3.3.1 WOMAC pain score

**FIGURE 3:**
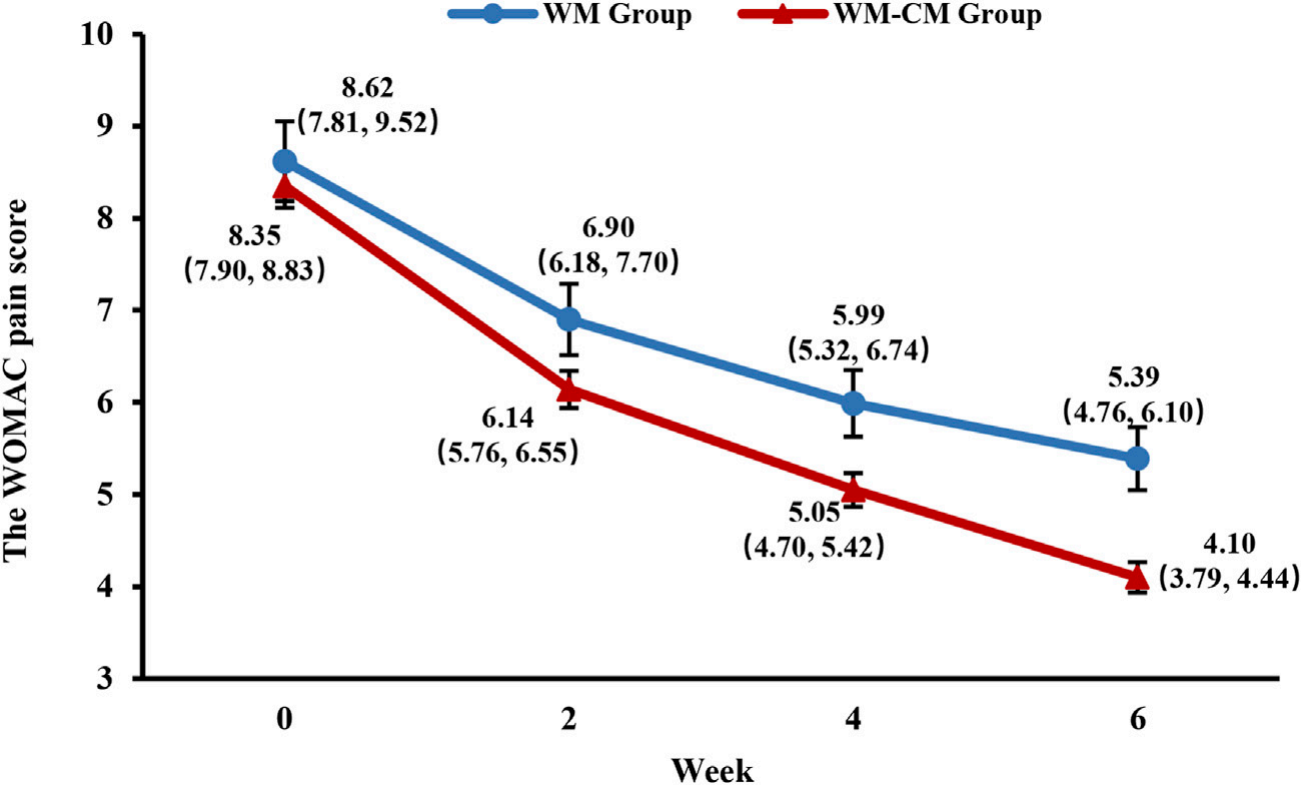
Overall trend of the WOMAC pain scores. Note: The values shown are least-squares mean calculated based on generalized linear mixed model, with 95% confidence intervals (indicated by error bars) in parentheses.

Figure 3 demonstrates the overall change in the WOMAC pain scores of both groups during the treatment period. With consistent baseline scores, the mean WOMAC pain score was significantly lower in the WM-CM group than in the WM group after 6 weeks of treatment (4.1 ± 0.165 vs. 5.39 ± 0.342, *p* = 0.001) (Table 4).

#### 3.3.2 WOMAC stiffness score

**FIGURE 4:**
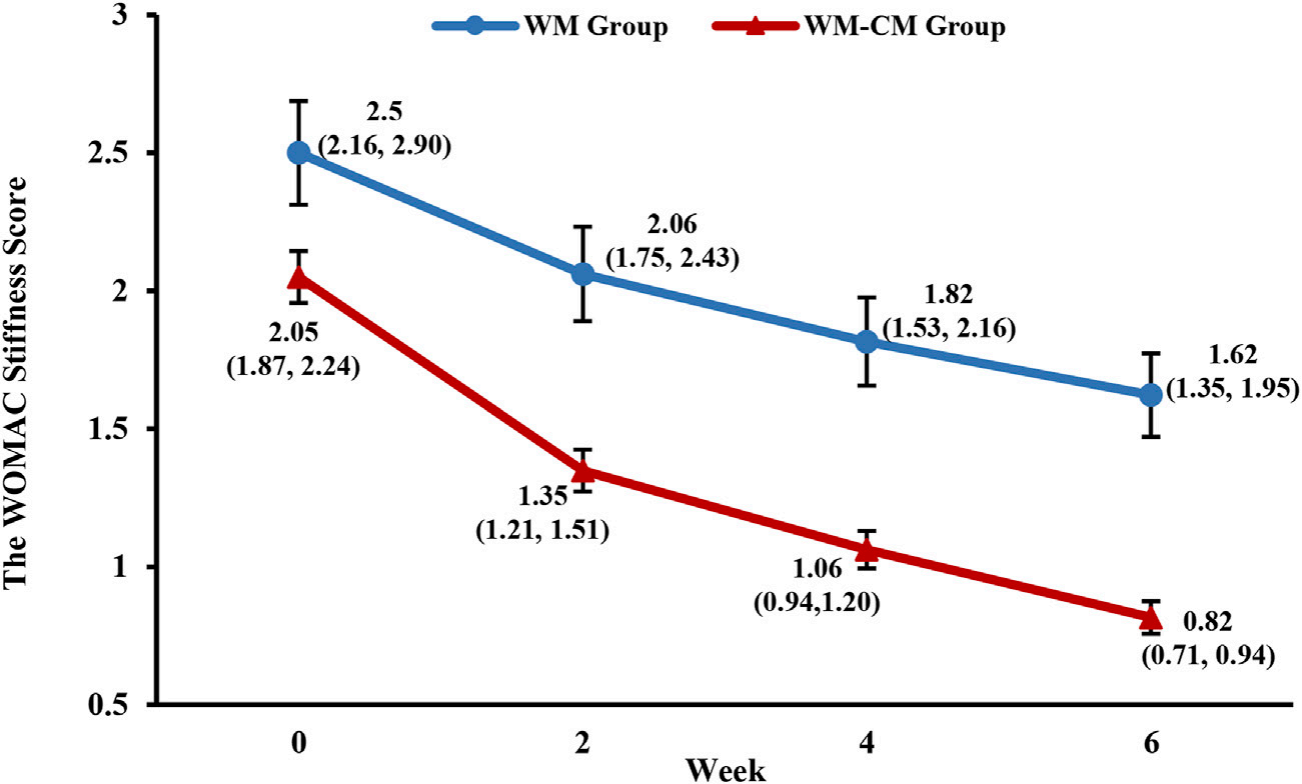
Overall trend of the WOMAC stiffness scores. Note: The values shown are least-squares mean calculated based on generalized linear mixed model, with 95% confidence intervals (indicated by error bars) in parentheses.

Figure 4 demonstrates the overall change in WOMAC stiffness scores over the treatment period for both groups. The large difference in baseline WOMAC stiffness scores between the two groups did not allow direct comparison of scores at 2, 4, and 6 weeks of treatment. Therefore, a comparison of the difference from baseline scores at 2, 4, and 6 weeks of treatment between the two groups was performed instead, and test scores were adjusted using the Bonferroni method. The results showed that the difference between the stiffness values at baseline and after treatment was slightly larger in the WM-CM group than in the WM group, but the difference was not statistically significant (*p* > 0.017) (Table 5).

**TABLE 5:** Comparison of the changes in WOMAC stiffness score from baseline.

	The changes in WOMAC stiffness score from baseline		*p*
	WM group	WM-CM group	
2 weeks	−0.44 ± 1.075	−0.70 ± 1.237	0.08
4 weeks	−0.68 ± 1.476	−0.99 ± 1.337	0.1
6 weeks	−0.88 ± 1.542	−1.23 ± 1.455	0.048

Note: The Mann‒Whitney U rank sum test was used for comparisons between groups, adjusted by the Bonferroni method, with a test level of 0.017.

#### 3.3.3 WOMAC physical function score

**FIGURE 5:**
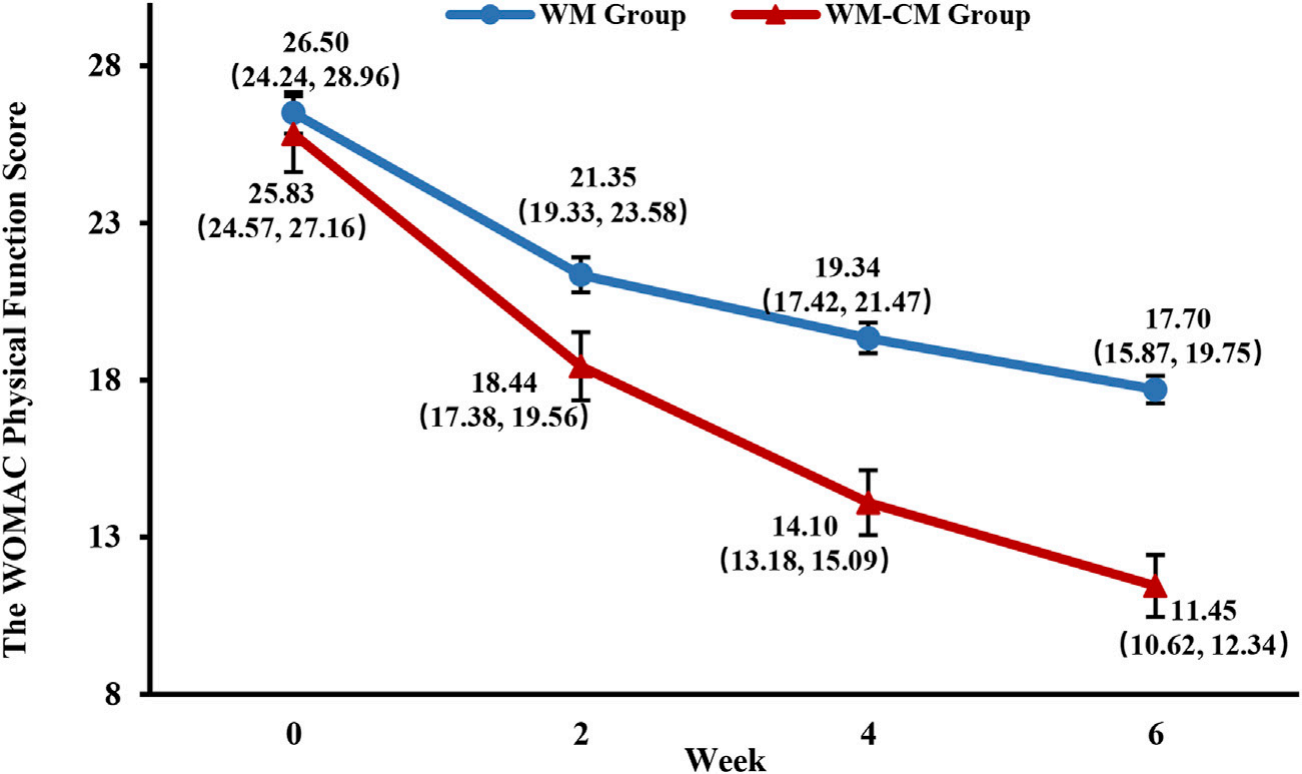
Overall trend of the WOMAC physical function scores. Note: The values shown are least-squares mean calculated based on generalized linear mixed model, with 95% confidence intervals (indicated by error bars) in parentheses.

Figure 5 demonstrates the overall changes in the WOMAC joint function scores of the two groups during the treatment period. With consistent baseline scores, the mean WOMAC joint function score was significantly lower in the WM-CM group than in the WM group after 6 weeks of treatment (11.45 ± 0.438 vs. 17.7 ± 0.986, *p* < 0.001) (Table 4).

#### 3.3.4 VAS score

**FIGURE 6:**
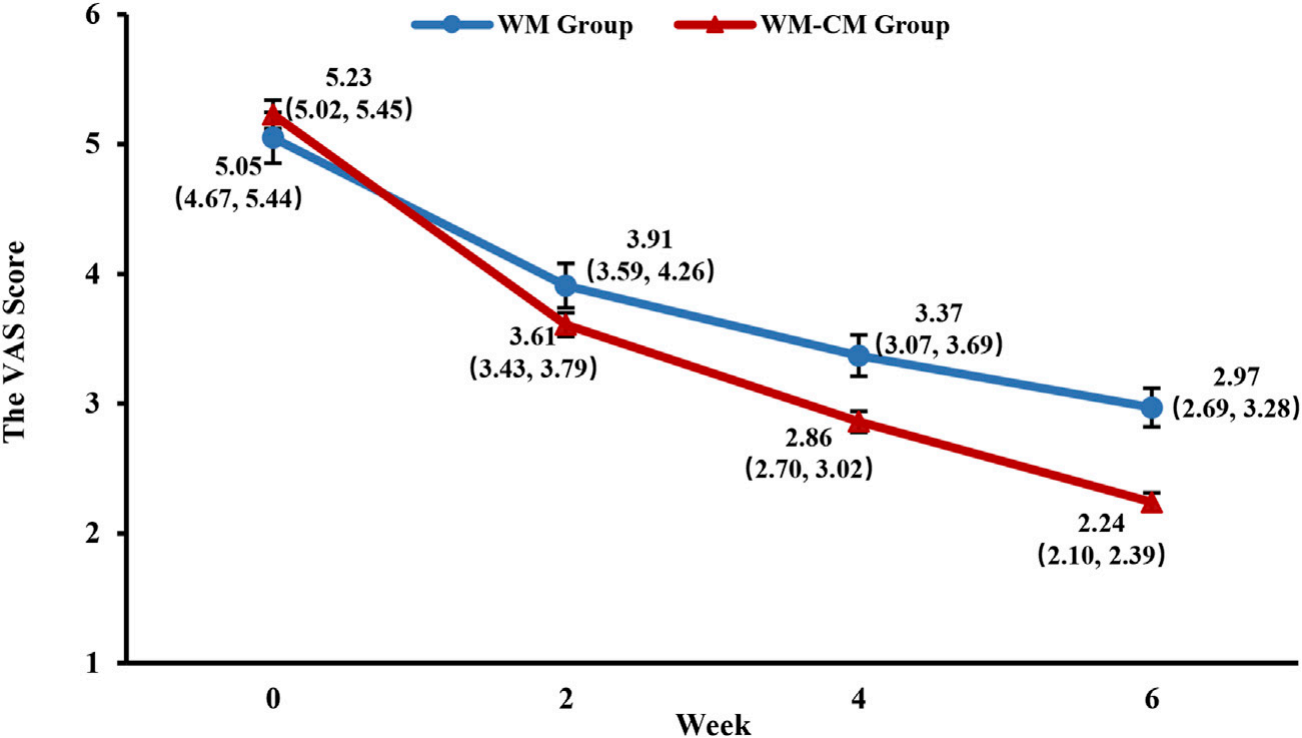
Overall trend of the VAS scores. Note: The values shown are least-squares mean calculated based on generalized linear mixed model, with 95% confidence intervals (indicated by error bars) in parentheses.

The VAS scores of both groups showed a significant decrease with the prolongation of treatment time, but the rate of decrease was faster in the WM-CM group (Figure 6). The intragroup and intergroup multiple comparisons of VAS scores between the two groups at each timepoint are detailed in Supplementary Table S3. After 6 weeks of treatment, the mean VAS score of the WM-CM group decreased to 2.24 ± 0.072, which was much lower than that of the WM group, which was 2.97 ± 0.149 (*p* < 0.001) (Table 4).

#### 3.3.5 PCS score

**FIGURE 7:**
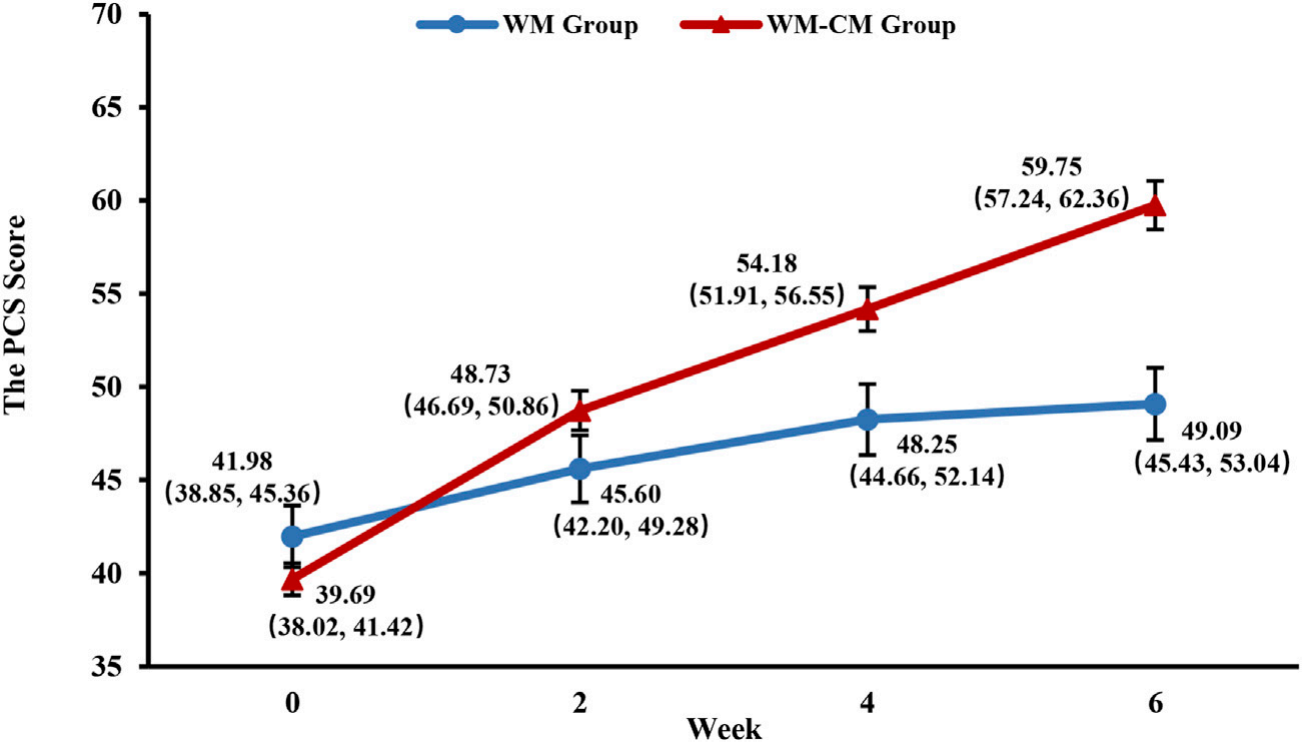
Overall trend of the PCS scores. Note: The values shown are least-squares mean calculated based on generalized linear mixed model, with 95% confidence intervals (indicated by error bars) in parentheses.

As shown in Figure 7, with consistent scores at baseline, the PCS scores of patients in the WM-CM group began to be gradually higher than those in the WM group after 2 weeks of treatment, but the difference was not statistically significant (*p* = 0.135). After 4 weeks of treatment, the PCS scores of patients in the WM-CM group were significantly higher than those in the WM group (*p* = 0.008), and after 6 weeks of treatment, the difference between the two groups was even more significant (*p* < 0.001) (Table 4; Supplementary Table S3). Details of the results of the 4 dimensions PF, RP, BP, and GH at each follow-up timepoint, as well as multiple comparisons within and between groups, are provided in the supplemental file.

#### 3.3.6 MCS score

**FIGURE 8:**
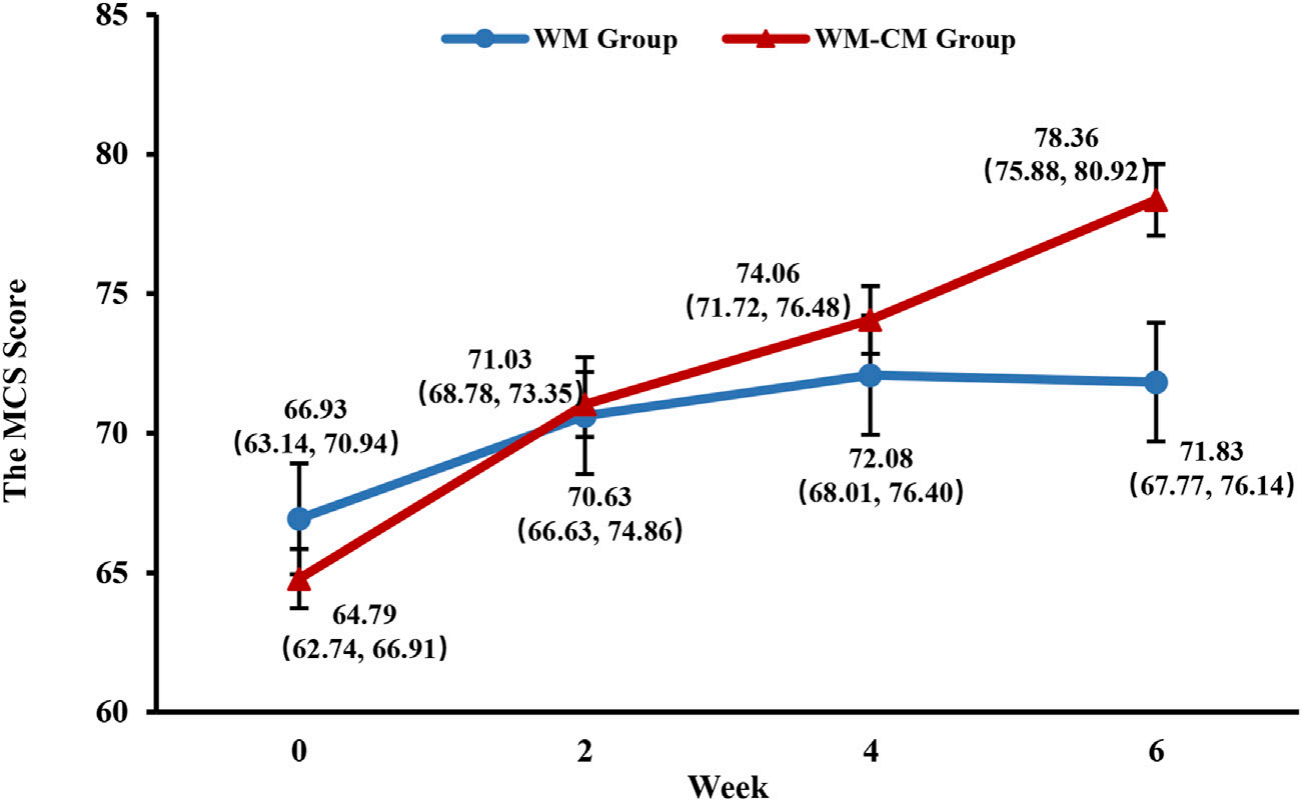
Overall trend of the MCS scores. Note: The values shown are least-squares mean calculated based on generalized linear mixed model, with 95% confidence intervals (indicated by error bars) in parentheses.

As shown in Figure 8, the MCS scores in the WM-CM group showed a steady upward trend with the prolongation of treatment time, and after 6 weeks of treatment, the mean MCS scores of the two groups began to show significant differences (*p* = 0.009) (Table 4). Details of the results of the 4 dimensions VT, SF, RE, and MH at each follow-up timepoint, as well as multiple comparisons within and between groups, are provided in the supplemental file.

#### 3.3.7 Clinical efficiency

As shown in Table 6, the effective rate of the WM-CM group was as high as 95%, which exceeded that of the WM group (80.6%), and the difference was statistically significant (*p* < 0.001); the significant response rate of the WM-CM group was 63.6%, which exceeded that of the WM group (32%), and the difference was statistically significant (*p* < 0.001).

**TABLE 6:** Comparison of clinical efficiency between groups.

	WM group (n = 98)	WM-CM group (n = 321)	$\chi^{2}$	*p*
effective	79 (80.6%)	305 (95%)	20.346	<0.001
significant	31 (32%)	204 (63.6%)	31.056	<0.001

### 3.4 Subgroup analysis

**FIGURE 9:**
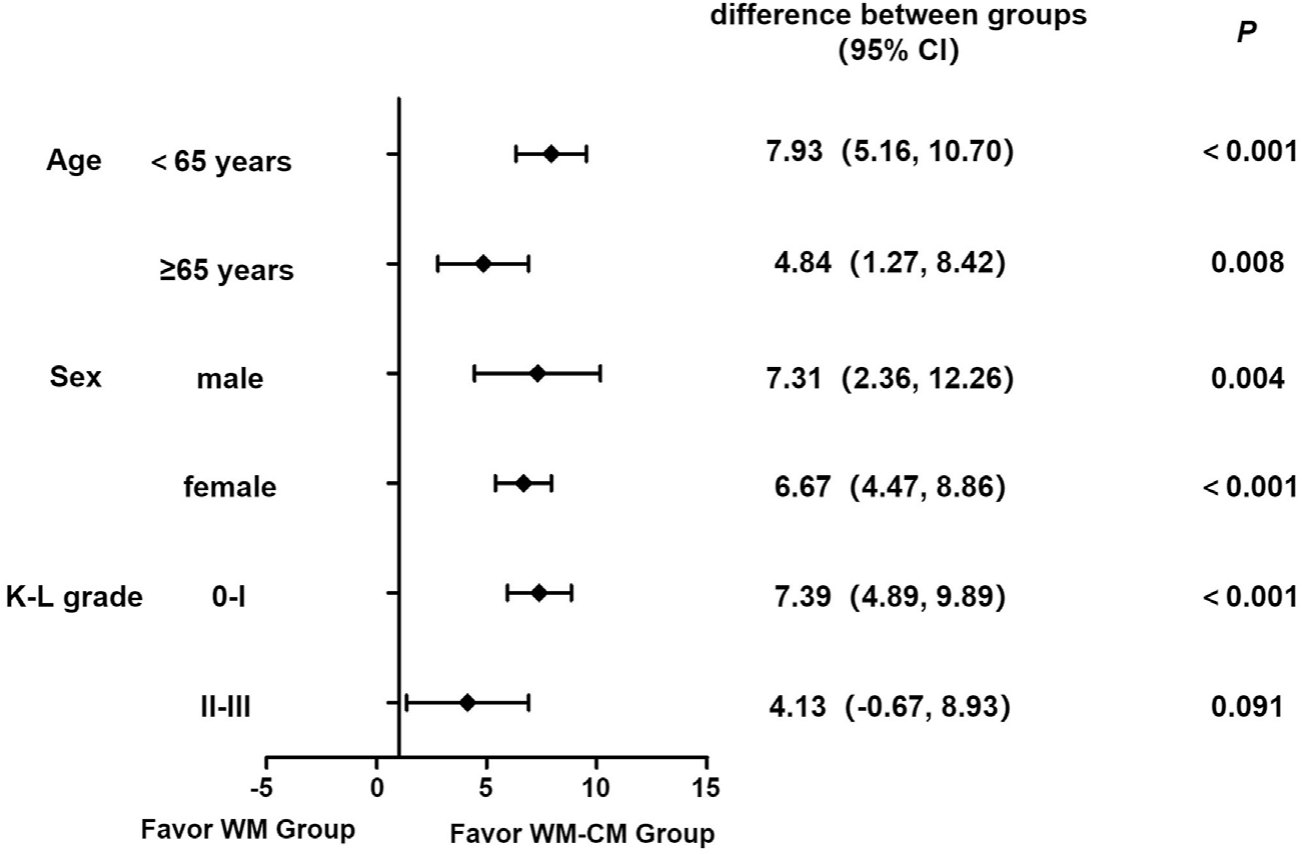
Forest plot for subgroup analysis. Note: The forest plot shows the differences in the changes in WOMAC total score between the two groups compared with baseline after 6 weeks of treatment stratifying by different subgroups. The difference between groups is calculated based on the least squares mean using GLMM, with a 95% confidence interval in parentheses. *p* values were Bonferroni corrected at a significance level of 0.05.

To examine the robustness of the results, we stratified patients by age, sex, and K-L grade and then compared the differences in changes in WOMAC total score between the two groups compared with baseline after 6 weeks of treatment in different subgroups using GLMM. As shown in Figure 9, in both the under-65 and over-65 age groups, the difference in WOMAC total score at baseline and after 6 weeks of treatment was larger in the WM-CM group than in the WM group, and the difference was significant (*p* < 0.001). The difference was more pronounced in the under-65 patient group. For both male and female patients, the improvement in WOMAC total score after 6 weeks of treatment was significantly better in the WM-CM group than in the WM group (*p* < 0.05). For patients with K-L classification 0-I, the WM-CM group performed better (*p* < 0.001), and for patients with grades II-III, the difference in WOMAC total score at baseline and after 6 weeks of treatment was slightly greater in the WM-CM group than in the WM group, but the difference was not significant (*p* = 0.091).

### 3.5 Safety evaluation

We focused on monitoring potential drug-related adverse events such as local pain and swelling from injections, gastrointestinal discomfort, cardiovascular events, and skin sensitization. No drug-related adverse events occurred in either group throughout the treatment cycle, and no patient discontinued the study due to adverse events.

## 4 Discussion

KOA has become a medical problem plaguing the world due to its high morbidity, high disability, high health hazard and high economic burden. Currently, Western drug treatment of KOA is based on Nonsteroidal Anti-inflammatory Drugs (NSAIDs), glucosamine, and sodium citrate injections. Among them, NSAIDs are recognized as first-line agents, but gastrointestinal and cardiovascular safety risks exist (Blumenthal K.G., 2017; Lim W.B., 2022), while the use of glucosamine and sodium citrate injections is controversial in clinical practice due to varying efficacy reported in different studies (Clegg D.O., 2006; Fransen M., 2015; Jevsevar D., 2015; Reginster J.Y., 2017; Honvo G., 2019; Peng B., 2019; Cai Z., 2022). Recently, there has been increasing evidence that TCM therapy is effective in preventing and treating KOA, improving clinical efficiency and reducing adverse effects (Zhu G.Q., 2014; Wu H.Y., 2015; Yu T. M, 2018), especially with Chinese patent medicines such as Xianling Gubao capsules, Zhuangguguanjie capsules, Jintiange capsules, Xiaotong patch and compound Nanxing analgesic ointment, which are recommended in many clinical guidelines in China (project group, 2021; Chen W.H., 2020; Association, 2021).

Most of the TCM preparations observed in this study have been proven to be effective in treating KOA in previous clinical researches (project group, 2021; Zhao J., 2022; Zhang W., 2016; Wu K.Y., 2015; Li X.L., 2019; Tan W.X., 2014; Guo X.X., 2017; Kang X.Z., 2011). However, it is important to note that these findings were obtained in the context of clinical trials and require verification in real-life clinical practice. The treatment process of TCM is highly individualized and complex, and the real medical environment is significantly more intricate than the controlled setting of a clinical trial. Therefore, real-world studies are essential to truly and comprehensively evaluate the effectiveness of complex TCM intervention programs. As far as we know, there have been very few large-scale real-world studies evaluating the overall efficacy of TCM combined with chemotherapy in the KOA field. Our early initiation of this research has the potential to garner attention from medical professionals, patients, and administrative personnel alike towards the benefits of TCM. We hope that the results of this research will receive the attention they deserve, enabling more KOA patients to benefit from the integration of TCM into their treatment plans.

In this study, we investigated the efficacy and safety of combined Chinese and Western medicine protocols for KOA patients with K-L score of 3 or less based on real clinical treatment scenarios. It was found that the combination of Chinese and Western medicine had significant advantages over Western medicine alone in relieving pain, improving knee function, and improving quality of life, and there was no significant difference between the two groups in relieving stiffness symptoms, but the interference caused by the insufficient reliability of the stiffness dimension of the WOMAC scale could not be excluded (Van de Graaf V.A., 2014), which needs to be further determined by combining other scales in future studies. In addition, subgroup analysis found that the benefit of the combination therapy group was more pronounced in younger patients under 65 years of age and in patients with milder disease with a K-L grade of 0-I. To some extent, this suggests an early intervention of TCM therapy.

The functions of the drugs used in this study are mainly to dispel wind and dampness, activate blood to relieve pain, and tonify the liver and kidney. “Jin-Gu theory” in TCM can make a reasonable explanation for why these drugs are selected in clinical practice. This theory regards KOA as a kind of “Jin-Gu” disease, and “Jin-Gu” refers to both the location of the disease and the different stages of the disease. When the disease is located in the “Jin”, it mainly affects the soft tissues around the knee. At this time, the disease is still in the early stage, which is an excess syndrome. It is necessary to use medicines for dispelling wind and dampness, and promoting blood circulation to soothe sinews and harmonize collaterals. When the disease is located in the “Gu”, the disease progresses and involves articular cartilage and bone tissue, and some symptoms of deficiency syndrome appear. Therefore, medicines for tonifying the liver and kidney are often chosen to protect cartilage and strengthen the bone.

Although most TCM doctors prescribe prescriptions based on TCM theory, the understanding of the active ingredients and targets of TCM is of great significance for elucidating the drug mechanism and even for the development of TCM. At present, studies have pointed out that Duhuo Jisheng Decoction and Yougui Pill can regulate the Wnt/β-catenin signaling pathway, thereby inhibiting the synovial inflammation and protecting articular cartilage (Lyu S., 2017; Yan C.L., 2018; Shi Q., 2022). Wangbi Tablet inhibits cartilage damage and inflammatory response by down-regulating NF-κB and p38-MAPK signaling pathways (Li H., 2021). The potential mechanism of Zhuifeng Tougu Pill against KOA may be the inhibition of TLR4/MyD88/NF-kB signaling pathway and inflammatory cytokines (Xu X., 2022). External preparations such as Wentong ointment and Daiwenjiu Ointment may promote the apoptosis of inflammatory cells, reduce the inflammatory response and inhibit neovascularization (Peng Y.L., 2014; Zhang L.W., 2022). Some botanical drugs frequently appear in these TCM prescriptions, showing immense potential in the treatment of KOA. Rehmanniae Radix Praeparata (Shu-Di-Huang) and Notopterygii Rhizoma Et Radix (Qiang-Huo) show significant anti-inflammatory effects (Pan T., 2017; Jhun J.Y., 2018). Drynariae Rhizoma (Gu-Sui-Bu) and its active components exhibit properties such as inhibiting inflammatory reactions, improving oxidative stress, suppressing cell apoptosis, regulating autophagy, influencing hormone levels, and enhancing microcirculation (Yang Y.J., 2021). Sodium ferulate, abundant in Angelicae Sinensis Radix (Dang-Gui), demonstrates remarkable anti-inflammatory and anti-apoptotic characteristics, while its polysaccharide component promotes the biosynthesis of proteoglycans in cartilage matrix (Magdalou J., 2015). Safflower yellow from Carthami Flos (Hong-Hua) protects chondrocytes and inhibits inflammation by regulating the NF-κB/SIRT1/AMPK pathway and ER stress (Wang C., 2020).

When selecting evaluation indicators, we refer to previous internationally influential literature and Chinese guidelines (Lu Z., 2018; Chen W.H., 2020; Belk J.W., 2021). In this study, the total score of WOMAC scale was used as the main efficacy index, which can comprehensively reflect the overall state of the knee joint of patients, and has high sensitivity and reliability for elderly patients and patients with mild symptoms (Walker L.C., 2018). The VAS score is mainly to quantify and visualize the pain symptoms of patients (Xu X.M., 2021), which is simpler and easier to understand than the WOMAC scale, and can be mutually corroborated with the WOMAC pain score. In addition, considering that chronic pain is significantly related to psychological depression and seriously affects the quality of life of patients, we also adopted the SF-36 scale, which is often used in combination with the WOMAC scale in the evaluation of the efficacy of KOA (Angst F., 2001).

We chose mature products that have been marketed for many years or in-hospital preparations developed from classical prescriptions or experimental prescriptions, and administered them following the recommended conventional methods stated in the instruction manual. This approach has three major advantages. Firstly, it provides clear side effect profiles, enabling us to better monitor adverse events and determine the correlation between adverse events and the medication. This study focused on monitoring all possible adverse events, and no drug-related adverse events occurred in either group throughout the treatment cycle. This strongly demonstrates the safety of short-term drug application as observed in our study. Secondly, the protocol is easy to replicate as the selected drugs are readily available, making it possible to repeat the study and apply the research plan more easily to a broader context. Thirdly, it reflects the real-world application of the medication, providing direct reference evidence for clinical treatment plan development.

Although this study controlled confounding factors through prospective design and statistical processing, it still has the common limitations of real-world studies. It is difficult to achieve randomization and blinding in real-world studies. Compared with randomized controlled trials, there are more confounding factors and bias. For example, our research center comprises two traditional Chinese medicine hospitals and one comprehensive hospital. Patients seen at the traditional Chinese medicine hospitals tend to lean towards receiving combined Chinese and Western medicine treatment, while patients visiting the comprehensive hospital are more inclined to undergo Western medicine treatment. Patient treatment preferences could introduce some bias into the study. In addition, the subjects of this study mainly come from three medical institutions, limiting the generalizability of the research conclusions to other populations. Therefore, cautious consideration is necessary when extrapolating the research conclusions to other populations. Moreover, due to the complexity of the intervention measures, the sample size currently included is still insufficient for conducting subgroup analysis of treatment regimens to further explore the differences in therapeutic effects among various approaches and their associations with disease phenotypes. Nonetheless, the results of our study do provide preliminary confirmation of the overall advantages of integrated Chinese and Western medicine in treating KOA. In the future, we will include more research centers and continue to increase the sample size to enhance the objectivity and rigor of our conclusions. The accumulation of research data will also facilitate further prescription analysis, helping us identify drugs with greater potential research value, leading to targeted randomized controlled trials and mechanism studies.

## 5 Conclusion

In summary, our findings demonstrate that TCM can be an important complementary therapy to conventional Western conservative treatment and should be used early and promptly. This conclusion still needs to be validated by further large cohort studies or randomized controlled trials.

## Data Availability

The original contributions presented in the study can be found in the article and in the Supplementary Material. The data underlying this article will be shared on reasonable request to the corresponding author.

## Abbreviations

KOA, knee osteoarthritis; WM group, Western medicine treatment group; WM-CM group, Combined Western and traditional Chinese medicine treatment group; PCS, Physical Component Summary; MCS, Mental Component Summary; WOMAC, Western Ontario and McMaster Universities Arthritis Index; VAS, the visual analogue scale; SF-36, 36-item short form; physical functioning; RP, physical functioning; BP, physical pain; CH, general health; VT, vitality; SF, social functioning; RE, emotional functioning; MH, mental health; GLMM, generalized linear mixed models; K-L, Kellgren-Lawrence; TCM, traditional Chinese medicine; NSAIDs, Nonsteroidal Antiinflammatory Drugs.
