# Supplementary material for: Efficacy and safety of combined Chinese and Western medicine in the treatment of knee osteoarthritis: a prospective, multicenter cohort study
Source: Front Pharmacol. 2023 Aug 28;14:1176980. doi: 10.3389/fphar.2023.1176980 (PMC10494435; doi:10.3389/fphar.2023.1176980)
Supplement: Supplementary file 1 [file DataSheet1.docx]

Supplementary Material

Table S1 Details of additional Chinese medicine prescriptions in this study

| **Medication Type** | **Ingredients** | **Percentage (%)** | **Processing** | **Approval Number** | **Executive Standard** | **Usage and Dosage** |
| --- | --- | --- | --- | --- | --- | --- |
| **Oral Chinese medicine** |  |  |  |  |  |  |
| Gulong Capsule* | dog bones (Gou-Tui-Gu) | 76.9 | Decoct the dog leg bone until the glue is exhausted. Add water to decoct Dioscorea nipponica Makino[Dioscorea; Dioscoreae Nipponicae Rhizoma(Chuan-Shan-Long)], combine the medicinal liquid and glue liquid, continue to concentrate until thick paste, dry, crush, and pack into capsules. | Z37021385 | The sixth volume of standard Chinese medicine prescriptions issued by the Ministry of Health and the national drug standard (revised) promulgation document, standard numberWS3-B-1181-92 | 4 capsules at a time, 3 times a day |
|  | Dioscorea nipponica Makino[Dioscorea; Dioscoreae Nipponicae Rhizoma(Chuan-Shan-Long)] | 23.1 |  |  |  |  |
| Wangbi Tablet* | Rehmannia glutinosa (Gaertn.) DC.[Orobanchaceae; Rehmanniae Radix(Di-Huang)] | 8.4 | Half the amount of Paeonia Lactiflora Pall.[Paeoniaceae; Paeoniae Radix Alba (Bai-Shao)] and Anemarrhena asphodeloides Bunge[Asparagaceae; Anemarrhenae Rhizoma(Zhi-Mu)] is crushed into fine powder, and the remaining drugs are decocted in batches, the liquid is concentrated under reduced pressure to the weight of the original drugs, and three times the amount of ethanol is added, and the extract is concentrated under reduced pressure to a relative density of 1.27~1.30 (50 °C) thick paste, mixed with powder to make granules, and then compressed into tablets. | Z20044066 | Chinese Pharmacopoeia 2020 Edition Part One | 4 tablets at a time, 3 times a day |
|  | Rehmannia Glutinosa (Gaertn.) DC.[Orobanchaceae; Rehmanniae Radix Praeparata (Shu-Di-Huang)] | 8.4 |  |  |  |  |
|  | Dipsacus asper Wall. ex DC.[Dipsacaceae; Dipsaci Radix(Xu-Duan)] | 6.3 |  |  |  |  |
|  | Aconitum carmichaelii Debeaux[Ranunculaceae; Aconiti Lateralis Radix Praeparata(Pao-Fu-Zi)] | 6.3 |  |  |  |  |
|  | Angelica Biserrata (R.H.Shan & C.Q.Yuan) C.Q.Yuan & R.H.Shan[Apiaceae; Angelicae Pubescentis Radix (Du-Huo)] | 4.2 |  |  |  |  |
|  | Drynaria roosii Nakaike[Polypodiaceae; Drynariae Rhizoma(Gu-Sui-Bu)] | 6.3 |  |  |  |  |
|  | Neolitsea Cassia (L.) Kosterm.[Lauraceae; Cinnamomi Ramulus (Gui-Zhi)] | 4.2 |  |  |  |  |
|  | Epimedium sagittatum (Siebold & Zucc.) Maxim.[Berberidaceae; Epimedii Folium(Yin-Yang-Huo)] | 6.3 |  |  |  |  |
|  | Saposhnikovia Divaricata (Turcz. Ex Ledeb.) Schischk.[Apiaceae; Saposhnikoviae Radix (Fang-Feng) ] | 4.2 |  |  |  |  |
|  | Clematis Chinensis Osbeck[Ranunculaceae; Clematidis Radix Et Rhizoma (Wei-Ling-Xian)] | 6.3 |  |  |  |  |
|  | Gleditsia sinensis Lam.[Fabaceae; Gleditsiae Spina(Zao-Jiao-Ci)] | 4.2 |  |  |  |  |
|  | sheep bones (Yang-Gu) | 8.4 |  |  |  |  |
|  | Paeonia Lactiflora Pall.[Paeoniaceae; Paeoniae Radix Alba (Bai-Shao)] | 5.1 |  |  |  |  |
|  | Cibotium barometz (L.) J.Sm.[Cyatheaceae; Cibotii Rhizoma(Zhi-Gou-Ji)] | 6.3 |  |  |  |  |
|  | Anemarrhena asphodeloides Bunge[Asparagaceae; Anemarrhenae Rhizoma(Zhi-Mu)] | 6.3 |  |  |  |  |
|  | Lycopodium clavatum L.[Lycopodiaceae; Lycopodii Herba(Shen-Jin-Cao)] | 4.2 |  |  |  |  |
|  | Carthamus Tinctorius L.[Asteraceae; Carthami Flos (Hong-Hua)] | 4.2 |  |  |  |  |
| Yougui Capsule* | Rehmannia Glutinosa (Gaertn.) Dc.[Orobanchaceae; Rehmanniae Radix Praeparata (Shu-Di-Huang)] | 21.1 | Cinnamomum verum J.Presl[Lauraceae; Cinnamomi Cortex(Rou-Gui)] and Angelica sinensis (Oliv.) Diels[Apiaceae; Angelicae Sinensis Radix (Dang-Gui)] are steamed to extract volatile oil, and the collected volatile oil is clathrated with β-cyclodextrin. The dregs and other drugs are decocted with water, concentrated with ethanol to form a thick paste, and dextrin is added to make an extract powder. The above-mentioned extract powder and β-cyclodextrin inclusion compound are mixed and packed into capsules. | Z20030134 | State Drug Administration Standards (Trial)WS-718（Z-146）-2001 | 4 capsules at a time, 3 times a day |
|  | Aconitum carmichaelii Debeaux[Ranunculaceae; Aconiti Lateralis Radix Praeparata(Pao-Fu-Zi)]） | 5.3 |  |  |  |  |
|  | Cinnamomum verum J.Presl[Lauraceae; Cinnamomi Cortex(Rou-Gui)] | 5.3 |  |  |  |  |
|  | Dioscorea oppositifolia L.[Dioscoreaceae; Dioscoreae Rhizoma(Shan-Yao)] | 10.5 |  |  |  |  |
|  | Cornus officinalis Siebold & Zucc.[Cornaceae; Corni Fructus(Zhi-Shan-Zhu-Yu)] | 7.9 |  |  |  |  |
|  | Cuscuta chinensis Lam.[Convolvulaceae; Cuscutae Semen(Tu-Si-Zi)] | 10.5 |  |  |  |  |
|  | Cervi Cornus Colla | 10.5 |  |  |  |  |
|  | Lycium barbarum L.[Solanaceae; Lycii Fructus(Gou-Qi-Zi)] | 10.5 |  |  |  |  |
|  | Angelica sinensis (Oliv.) Diels[Apiaceae; Angelicae Sinensis Radix (Dang-Gui)] | 7.9 |  |  |  |  |
|  | Eucommia Ulmoides Oliv.[Eucommiaceae; Eucommiae Cortex (Yan-Du-Zhong)] | 10.5 |  |  |  |  |
| Zhongtongan Capsule^#^ | Panax notoginseng (Burkill) F.H.Chen[Araliaceae; Notoginseng Radix Et Rhizoma(San-Qi)] |  | All drugs are pulverized, mixed, dried and made into granules, and then filled into capsules. | Z13021496 | National Drug Standards WS3-B-3872-98-2005 | 2 capsules at a time, 3 times a day |
|  | Gastrodia elata Blume[Orchidaceae; Gastrodiae Rhizoma(Tian-Ma)] |  |  |  |  |  |
|  | Bombyx Batryticatus (Chao-Jiang-Can) |  |  |  |  |  |
|  | Sauromatum giganteum (Engl.) Cusimano & Hett.[Araceae; Typhonii Rhizoma(Zhi-Bai-Fu-Zi)] |  |  |  |  |  |
|  | Saposhnikovia Divaricata (Turcz. Ex Ledeb.) Schischk.[Apiaceae; Saposhnikoviae Radix (Fang-Feng) ] |  |  |  |  |  |
|  | Hansenia weberbaueriana (Fedde ex H.Wolff) Pimenov & Kljuykov[Apiaceae; Notopterygii Rhizoma Et Radix(Qiang-Huo)] |  |  |  |  |  |
|  | Arisaema erubescens (Wall.) Schott[Araceae; Arisaematis Rhizoma(Zhi-Tian-Nan-Xing)] |  |  |  |  |  |
|  | Angelica dahurica (Hoffm.) Benth. & Hook.f. ex Franch. & Sav.[Apiaceae; Angelicae Dahuricae Radix(Bai-Zhi)] |  |  |  |  |  |
| Zhuifeng Tougu Capsule* | Aconitum carmichaelii Debeaux[Ranunculaceae; Aconiti Radix(Zhi-Chuan-Wu)] | 5.2 | Asarum Heterotropoides F.Schmidt[Aristolochiaceae; Asari Radix Et Rhizome (Xi-Xin)] is distilled to extract aromatic oil. The remaining drugs are decocted with water, mixed with Asarum Heterotropoides F.Schmidt[Aristolochiaceae; Asari Radix Et Rhizome (Xi-Xin)] decoction liquid, and dried into powder. The powder is sprayed with Asarum Heterotropoides F.Schmidt[Aristolochiaceae; Asari Radix Et Rhizome (Xi-Xin)] volatile oil and then packed into capsules. | Z20173002 | State Food and Drug Administration Drug Standards YBZ04062008 | 4 capsules at a time, 2 times a day |
|  | Cyperus Rotundus L.[Cyperaceae; Cyperi Rhizoma(Zhi-Xiang-Fu)] | 5.2 |  |  |  |  |
|  | Conioselinum Anthriscoides 'Chuanxiong'[Apiaceae; Chuanxiong Rhizome (Chuan-Xiong)] | 5.2 |  |  |  |  |
|  | Ephedra sinica Stapf[Ephedraceae; Ephedrae Herba(Ma-Huang)] | 5.2 |  |  |  |  |
|  | Aconitum kusnezoffii Rchb.[Ranunculaceae; Aconiti Kusnezoffii Radix(Zhi-Cao-Wu)] | 5.2 |  |  |  |  |
|  | Gentiana Macrophylla Pall.[Gentianaceae; Gentianae Macrophyllae Radix (Qin-Jiao) ] | 2.6 |  |  |  |  |
|  | Angelica sinensis (Oliv.) Diels[Apiaceae; Angelicae Sinensis Radix (Dang-Gui)] | 2.6 |  |  |  |  |
|  | Vigna umbellata (Thunb.) Ohwi & H.Ohashi[Fabaceae; Vignae Semen(Chi-Xiao-Dou)] | 5.2 |  |  |  |  |
|  | Hansenia weberbaueriana (Fedde ex H.Wolff) Pimenov & Kljuykov[Apiaceae; Notopterygii Rhizoma Et Radix(Qiang-Huo)] | 5.2 |  |  |  |  |
|  | Paeonia lactiflora Pall.[Paeoniaceae; Paeoniae Radix Rubra(Chi-Shao)] | 5.2 |  |  |  |  |
|  | Asarum Heterotropoides F.Schmidt[Aristolochiaceae; Asari Radix Et Rhizome (Xi-Xin)] | 5.2 |  |  |  |  |
|  | Arisaema erubescens (Wall.) Schott[Araceae; Arisaematis Rhizoma(Zhi-Tian-Nan-Xing)] | 5.2 |  |  |  |  |
|  | Angelica dahurica (Hoffm.) Benth. & Hook.f. ex Franch. & Sav.[Apiaceae; Angelicae Dahuricae Radix(Bai-Zhi)] | 5.2 |  |  |  |  |
|  | Glycyrrhiza Glabra L.[Fabaceae; Glycyrrhizae Radix Et Rhizoma (Gan-Cao)] | 5.2 |  |  |  |  |
|  | Atractylodes macrocephala Koidz.[Asteraceae; Atractylodis Macrocephalae Rhizoma(Chao-Bai-Zhu)] | 2.6 |  |  |  |  |
|  | Commiphora Myrrha (T.Nees) Engl.[Burseraceae; Myrrha(Zhi-Mo-Yao)] | 1.0 |  |  |  |  |
|  | Boswellia Sacra Flück. [Burseraceae; Olibanum (Zhi-Ru-Xiang)] | 2.6 |  |  |  |  |
|  | Pheretima (Di-Long) | 5.2 |  |  |  |  |
|  | Carapichea Ipecacuanha (Brot.) L.Andersson[Polyporaceae; Poria (Fu-Ling)] | 10.4 |  |  |  |  |
|  | Neolitsea Cassia (L.) Kosterm.[Lauraceae; Cinnamomi Ramulus (Gui-Zhi)] | 2.6 |  |  |  |  |
|  | Gastrodia elata Blume[Orchidaceae; Gastrodiae Rhizoma(Tian-Ma)] | 2.6 |  |  |  |  |
|  | Nardostachys jatamansi (D.Don) DC.[Caprifoliaceae, Nardostachyos Radix Et Rhizoma(Gan-Song)] | 2.6 |  |  |  |  |
|  | Saposhnikovia Divaricata (Turcz. Ex Ledeb.) Schischk.[ Apiaceae; Saposhnikoviae Radix (Fang-Feng) ] | 2.6 |  |  |  |  |
| Hugu Capsule^#^ | Reynoutria multiflora (Thunb.) Moldenke[Polygonaceae; Polygoni Multiflori Radix(Zhi-He-Shou-Wu)] | 12.5 | Take 1/3 amount of Dioscorea oppositifolia L.[Dioscoreaceae; Dioscoreae Rhizoma(Shan-Yao)] and crush it into powder. The remaining drugs are decocted with water and the volatile oil is collected. The water decoction is spray-dried to form a dry cream powder. The volatile oil is dissolved in a small amount of ethanol and sprayed into the above Shan-Yao powder, then mixed with the dry cream powder, and packed into capsules. | Z20040124 | State Food and Drug Administration National Drug StandardsYBZ12742004-2009Z | 4 capsules each time, three times a day |
|  | Epimedium sagittatum (Siebold & Zucc.) Maxim.[Berberidaceae; Epimedii Folium(Yin-Yang-Huo)] | 10.0 |  |  |  |  |
|  | Rehmannia Glutinosa (Gaertn.) Dc.[Orobanchaceae; Rehmanniae Radix Praeparata (Shu-Di-Huang)] | 12.5 |  |  |  |  |
|  | Testudinis Carapax Et Plastrum (Gui-Jia) | 7.5 |  |  |  |  |
|  | Gynochthodes officinalis (F.C.How) Razafim. & B.Bremer[Rubiaceae; Morindae Officinalis Radix(Ba-Ji-Tian)] | 10.0 |  |  |  |  |
|  | Eucommia Ulmoides Oliv.[Eucommiaceae; Eucommiae Cortex (Du-Zhong)] | 10.0 |  |  |  |  |
|  | Dipsacus asper Wall. ex DC.[Dipsacaceae; Dipsaci Radix(Xu-Duan)] | 10.0 |  |  |  |  |
|  | Drynaria roosii Nakaike[Polypodiaceae; Drynariae Rhizoma(Gu-Sui-Bu)] | 10.0 |  |  |  |  |
|  | Angelica sinensis (Oliv.) Diels[Apiaceae; Angelicae Sinensis Radix (Dang-Gui)] | 7.5 |  |  |  |  |
|  | Dioscorea oppositifolia L.[Dioscoreaceae; Dioscoreae Rhizoma(Shan-Yao)] | 10.0 |  |  |  |  |
| Tenghuang Jiangu Tablet^#^ | Rehmannia Glutinosa (Gaertn.) Dc.[Orobanchaceae; Rehmanniae Radix Praeparata (Shu-Di-Huang)] | 21.4 | Pyrola calliantha Andres[Ericaceae; Pyrolae Herba(Lu-Xian-Cao)] and Epimedium sagittatum (Siebold & Zucc.) Maxim.[Berberidaceae; Epimedii Folium(Yin-Yang-Huo)] are crushed into fine powder. The remaining drugs are decocted with water. The filtrate is condensed to a thick paste, mixed with the above powder, dried, pulverized, and then made into tablets. | Z20090570 | State Food and Drug Administration Drug Standards YBZ07942009 | 4 tablets at a time, 2 times a day |
|  | Pyrola calliantha Andres[Ericaceae; Pyrolae Herba(Lu-Xian-Cao)] | 14.3 |  |  |  |  |
|  | Drynaria roosii Nakaike[Polypodiaceae; Drynariae Rhizoma(Gu-Sui-Bu)] | 14.3 |  |  |  |  |
|  | Cistanche deserticola Ma[Orobanchaceae; Cistanches Herba(Rou-Cong-Rong)] | 14.3 |  |  |  |  |
|  | Epimedium sagittatum (Siebold & Zucc.) Maxim.[Berberidaceae; Epimedii Folium(Yin-Yang-Huo)] | 14.3 |  |  |  |  |
|  | Spatholobus suberectus Dunn[Fabaceae; Spatholobi Caulis(Ji-Xue-Teng)] | 14.3 |  |  |  |  |
|  | Raphanus raphanistrum subsp. sativus (L.) Domin[Brassicaceae; Raphani Semen(Lai-Fu-Zi)] | 7.1 |  |  |  |  |
| Dahuoluo Capsule^#^ | Panax ginseng C.A.Mey.[Araliaceae; Ginseng Radix Et Rhizoma Rubra(Hong-Shen)] | 4.4 | The drugs are crushed into fine powder, sprayed with a suspension of Moschus (Ren-Gong-She-Xiang), Borneolum Syntheticum (Bing-Pian) and ethanol, and made into capsules. | Z19990044 | State Food and Drug Administration National Drug Standards WS3-304（Z-32）-2003（Z） | 4 capsules at a time, 3 times a day |
|  | Atractylodes macrocephala Koidz.[Asteraceae; Atractylodis Macrocephalae Rhizoma(Chao-Bai-Zhu)] | 1.5 |  |  |  |  |
|  | Glycyrrhiza Glabra L.[Fabaceae; Glycyrrhizae Radix Et Rhizoma (Gan-Cao)] | 3.0 |  |  |  |  |
|  | Rehmannia Glutinosa (Gaertn.) Dc.[Orobanchaceae; Rehmanniae Radix Praeparata (Shu-Di-Huang)] | 3.0 |  |  |  |  |
|  | Angelica sinensis (Oliv.) Diels[Apiaceae; Angelicae Sinensis Radix (Dang-Gui)] | 2.2 |  |  |  |  |
|  | Reynoutria multiflora (Thunb.) Moldenke[Polygonaceae; Polygoni Multiflori Radix(He-Shou-Wu)] | 3.0 |  |  |  |  |
|  | Testudinis Carapax Et Plastrum (Cu-Gui-Jia) | 1.0 |  |  |  |  |
|  | Boswellia Sacra Flück. [Olivaceae; Olibanum (Zhi-Ru-Xiang) ] | 1.5 |  |  |  |  |
|  | Commiphora Myrrha (T.Nees) Engl.[Burseraceae; Myrrha(Zhi-Mo-Yao)] | 1.5 |  |  |  |  |
|  | Calamus draco Willd.[Arecaceae; Draconis Sanguis(Xue-Jie)] | 1.0 |  |  |  |  |
|  | Paeonia lactiflora Pall.[Paeoniaceae; Paeoniae Radix Rubra(Chi-Shao)] | 1.5 |  |  |  |  |
|  | Cinnamomum verum J.Presl[Lauraceae; Cinnamomi Cortex(Rou-Gui)] | 3.0 |  |  |  |  |
|  | Anemonoides raddeana (Regel) Holub[Ranunculaceae; Anemones Raddeanae Rhizoma(Liang-Tou-Jian)] | 3.0 |  |  |  |  |
|  | Moschus (Ren-Gong-She-Xiang) | 0.8 |  |  |  |  |
|  | Borneolum Syntheticum (Bing-Pian) | 0.2 |  |  |  |  |
|  | Styrax benzoin Dryand.[Styracaceae; Benzoinum(An-Xi-Xiang)] | 1.5 |  |  |  |  |
|  | Aquilaria sinensis (Lour.) Spreng.[Thymelaeaceae; Aquilariae Lignum Resinatum(Chen-Xiang)] | 3.0 |  |  |  |  |
|  | Dolomiaea costus (Falc.) Kasana & A.K.Pandey[Asteraceae; Aucklandiae Radix(Mu-Xiang)] | 3.0 |  |  |  |  |
|  | Syzygium aromaticum (L.) Merr. & L.M.Perry[Myrtaceae; Caryophylli Flos(Ding-Xiang)] | 1.5 |  |  |  |  |
|  | Cyperus Rotundus L.[Cyperaceae; Cyperi Rhizoma(Cu-Xiang-Fu)] | 1.5 |  |  |  |  |
|  | Powerdered Buffalo Horn Extract Extract (Shui-Niu-Jiao Nong-Suo-Fen) | 0.8 |  |  |  |  |
|  | Lindera aggregata (Sims) Kosterm.[Lauraceae; Linderae Radix(Wu-Yao)] | 3.0 |  |  |  |  |
|  | Citrus × aurantium f. deliciosa (Ten.) M.Hiroe[Rutaceae; Citri Reticulatae Pericarpium Viride(Qing-Pi)] | 1.5 |  |  |  |  |
|  | Aconitum kusnezoffii Rchb.[Ranunculaceae; Aconiti Kusnezoffii Radix(Zhi-Cao-Wu)] | 3.0 |  |  |  |  |
|  | Ephedra sinica Stapf[Ephedraceae; Ephedrae Herba(Ma-Huang)] | 3.0 |  |  |  |  |
|  | Asarum Heterotropoides F.Schmidt[Aristolochiaceae; Asari Radix Et Rhizome (Xi-Xin)] | 1.5 |  |  |  |  |
|  | Hansenia weberbaueriana (Fedde ex H.Wolff) Pimenov & Kljuykov[Apiaceae; Notopterygii Rhizoma Et Radix(Qiang-Huo)] | 1.5 |  |  |  |  |
|  | Saposhnikovia Divaricata (Turcz. Ex Ledeb.) Schischk.[ Apiaceae; Saposhnikoviae Radix (Fang-Feng) ] | 3.7 |  |  |  |  |
|  | Agkistrodon (Qi-She) | 3.0 |  |  |  |  |
|  | Zaocys (Wu-Shao-She) | 3.0 |  |  |  |  |
|  | Leopard Bone (Bao-Gu) | 2.2 |  |  |  |  |
|  | Hypericum perforatum L.[Hypericaceae; Rosin(Song-Xiang)] | 0.8 |  |  |  |  |
|  | Drynaria roosii Nakaike[Polypodiaceae; Drynariae Rhizoma(Gu-Sui-Bu)] | 1.5 |  |  |  |  |
|  | Gastrodia elata Blume[Orchidaceae; Gastrodiae Rhizoma(Tian-Ma)] | 3.0 |  |  |  |  |
|  | Arisaema erubescens (Wall.) Schott[Araceae; Arisaematis Rhizoma(Zhi-Tian-Nan-Xing)] | 1.5 |  |  |  |  |
|  | Scorpio (Quan-Xie)] | 3.0 |  |  |  |  |
|  | Bombyx Batryticatus (Chao-Jiang-Can) | 1.5 |  |  |  |  |
|  | Pheretima (Di-Long)] | 0.8 |  |  |  |  |
|  | Pueraria montana var. lobata (Willd.) Maesen & S.M.Almeida ex Sanjappa & Predeep[Fabaceae; Puerariae Lobatae Radix(Ge-Gen)] | 2.2 |  |  |  |  |
|  | Wurfbainia compacta (Sol. ex Maton) Škorničk. & A.D.Poulsen[Zingiberaceae; Amomi Fructus Rotundus (Dou-Kou)] | 1.5 |  |  |  |  |
|  | Pogostemon cablin (Blanco) Benth.[Lamiaceae; Pogostemonis Herba(Guang-Huo-Xiang)] | 3.0 |  |  |  |  |
|  | Dryopteris crassirhizoma Nakai[Polypodiaceae; Dryopteridis Crassirhizomatis Rhizoma(Mian-Ma-Guan-Zhong)] | 3.0 |  |  |  |  |
|  | Bovis Calculus Artifactus (Ren-Gong Niu-Huang)] | 0.2 |  |  |  |  |
|  | Rheum officinale Baill.[Polygonaceae; Rhei Radix Et Rhizoma(Da-Huang)] | 3.0 |  |  |  |  |
|  | Coptis chinensis Franch.[Ranunculaceae; Coptidis Rhizoma(Huang-Lian)] | 3.0 |  |  |  |  |
|  | Scutellaria baicalensis Georgi[Lamiaceae; Scutellariae Radix(Huang-Qin)] | 1.5 |  |  |  |  |
|  | Scrophularia ningpoensis Hemsl.[Scrophulariaceae; Scrophulariae Radix(Xuan-Shen)] | 1.5 |  |  |  |  |
|  | Clematis Chinensis Osbeck[Ranunculaceae; Clematidis Radix Et Rhizoma (Wei-Ling-Xian)] | 3.0 |  |  |  |  |
| Xianling Gubao Capsule^#^ | Epimedium sagittatum (Siebold & Zucc.) Maxim.[Berberidaceae; Epimedii Folium(Yin-Yang-Huo)] | 70.0 | Dipsacus asper Wall. ex DC.[Dipsacaceae; Dipsaci Radix(Xu-Duan)], Salvia Miltiorrhiza Bunge[Labiatae; Salviae Miltiorrhizae Radix Et Rhizoma (Dan-Shen)], and Cullen corylifolium (L.) Medik.[Fabaceae; Psoraleae Fructus(Bu-Gu-Zhi)] are crushed into fine powder. The rest of the drugs are decocted to make a thick paste, and the above powder is added, mixed evenly, dried, pulverized, and packed into capsules. | Z20025337 | State Food and Drug Administration National Drug Standards WS-10269（ZD-0269）-2002-2011Z | 3 capsules at a time, 2 times a day |
|  | Dipsacus asper Wall. ex DC.[Dipsacaceae; Dipsaci Radix(Xu-Duan)] | 10.0 |  |  |  |  |
|  | Salvia Miltiorrhiza Bunge[Labiatae; Salviae Miltiorrhizae Radix Et Rhizoma (Dan-Shen)] | 5.0 |  |  |  |  |
|  | Anemarrhena asphodeloides Bunge[Asparagaceae; Anemarrhenae Rhizoma(Zhi-Mu)] | 5.0 |  |  |  |  |
|  | Cullen corylifolium (L.) Medik.[Fabaceae; Psoraleae Fructus(Bu-Gu-Zhi)] | 5.0 |  |  |  |  |
|  | Rehmannia glutinosa (Gaertn.) DC.[Orobanchaceae; Rehmanniae Radix(Di-Huang)] | 5.0 |  |  |  |  |
| **External Chinese medicine** |  |  |  |  |  |  |
| Cooling Blood Swelling Ointment^#^ | Gypsum Ustum (Duan-Shi-Gao) |  | The drug is crushed into fine powder, and the auxiliary materials are added to make ointment. | Z20180005  (Guangdong batch number) | Registration Standards for Preparations in Medical Institutions | 1 to 2 times a day |
| Cooling Jingjintong Plaster^#^ | Phellodendron amurense Rupr.[Rutaceae; Phellodendri Amurensis Cortex(Guan-Huang-Bo) |  | The drugs are crushed into fine powder, and the auxiliary materials are added to make ointment. | Z20140010  (Guangdong batch number) | Registration Standards for Preparations in Medical Institutions | 24 hours per patch |
|  | Rheum officinale Baill.[Polygonaceae; Rhei Radix Et Rhizoma(Da-Huang)] |  |  |  |  |  |
|  | Zanthoxylum nitidum (Roxb.) DC.[Rutaceae; Zanthoxyli Radix(Liang-Mian-Zhen)] |  |  |  |  |  |
|  | Paeonia × suffruticosa Andrews[Paeoniaceae; Moutan Cortex(Mu-Dan-Pi)] |  |  |  |  |  |
|  | Gardenia jasminoides J.Ellis[Rubiaceae; Gardeniae Fructus(Zhi-Zi)] |  |  |  |  |  |
|  | Carthamus Tinctorius L.[Asteraceae; Carthami Flos (Hong-Hua)] |  |  |  |  |  |
| Wentong Ointment^$^ | Cullen corylifolium (L.) Medik.[Fabaceae; Psoraleae Fructus(Bu-Gu-Zhi)] | 6.9 | The drugs are extracted with ethanol to make a clear paste. Add carbomer to the clear paste and soak for 24 hours, then add Camphor(Racemic) (Zhang-Nao(He-Cheng)), Borneolum Syntheticum (Bing-Pian), L-Menthol (Bo-He-Nao), Turpentine Oil (Song-Jie You), Methyl salicylate, and mix well. | Z20080086  (Guangdong batch number) | Registration Standards for Preparations in Medical Institutions | 3 to 4 times a day |
|  | Aconitum carmichaelii Debeaux[Ranunculaceae; Aconiti Lateralis Radix Praeparata(Fu-Zi)] | 1.4 |  |  |  |  |
|  | Cnidium monnieri (L.) Cusson[Apiaceae; Cnidii Fructus(She-Chuang-Zi)] | 5.5 |  |  |  |  |
|  | Dipsacus asper Wall. ex DC.[Dipsacaceae; Dipsaci Radix(Xu-Duan)] | 6.9 |  |  |  |  |
|  | Rubus chingii Hu[Rosaceae; Rubi Fructus(Fu-Pen-Zi)] | 6.9 |  |  |  |  |
|  | Styrax benzoin Dryand.[Styracaceae; Benzoinum(An-Xi-Xiang)] | 1.4 |  |  |  |  |
|  | Zingiber officinale Roscoe[Zingiberaceae; Zingiberis Rhizoma(Gan-Jiang)] | 2.7 |  |  |  |  |
|  | Capsicum annuum L.[Solanaceae; Capsici Fructus(La-Jiao)] | 5.5 |  |  |  |  |
|  | Polygala senega L.[Polygalaceae; Polygalae Radix(Yuan-zhi)] | 6.9 |  |  |  |  |
|  | Cibotium barometz (L.) J.Sm.[Cyatheaceae; Cibotii Rhizoma(Gou-Ji)] | 6.9 |  |  |  |  |
|  | Aconitum carmichaelii Debeaux[Ranunculaceae; Aconiti Radix(Chuan-Wu)] | 13.7 |  |  |  |  |
|  | Angelica sinensis (Oliv.) Diels[Apiaceae; Angelicae Sinensis Radix (Dang-Gui)] | 6.9 |  |  |  |  |
|  | Hansenia weberbaueriana (Fedde ex H.Wolff) Pimenov & Kljuykov[Apiaceae; Notopterygii Rhizoma Et Radix(Qiang-Huo)] | 5.5 |  |  |  |  |
|  | Angelica Biserrata (R.H.Shan & C.Q.Yuan) C.Q.Yuan & R.H.Shan[Apiaceae; Angelicae Pubescentis Radix (Du-Huo) ] | 5.5 |  |  |  |  |
|  | Alpinia officinarum Hance[Zingiberaceae; Alpiniae Officinarum Rhizoma(Gao-Liang-Jiang)] | 2.7 |  |  |  |  |
|  | Arisaema erubescens (Wall.) Schott[Araceae; Arisaematis Rhizoma(Tian-Nan-Xing)] | 13.7 |  |  |  |  |
|  | Syzygium aromaticum (L.) Merr. & L.M.Perry[Myrtaceae; Caryophylli Flos(Ding-Xiang)] | 1.4 |  |  |  |  |
| Li Guanghai Dieda Qufeng Ointment^#^ | Panax notoginseng (Burkill) F.H.Chen[Araliaceae; Notoginseng Radix Et Rhizoma(San-Qi)] |  | The drugs are crushed into fine powder, and the auxiliary materials are added to make ointment. | Z03140142  (Guangdong batch number) | Registration Standards for Preparations in Medical Institutions | external use |
|  | Arisaema erubescens (Wall.) Schott[Araceae; Arisaematis Rhizoma(Tian-Nan-Xing)] |  |  |  |  |  |
|  | Actaea racemosa L.[Ranunculaceae; Cimicifugae Rhizoma(Sheng-Ma)] |  |  |  |  |  |
| Tianbai Golden Plaster^$^ | Trichosanthes kirilowii Maxim.[Cucurbitaceae; Trichosanthis Radix(Tian-Hua-Fen) | 22.7 | The drugs are crushed into fine powder and mix well. | Z20190014000  (Guangdong batch number) | Registration Standards for Preparations in Medical Institutions | powder into paste, external application |
|  | Phellodendron amurense Rupr.[Rutaceae; Phellodendri Amurensis Cortex(Huang-Bo) | 11.3 |  |  |  |  |
|  | Rheum officinale Baill.[Polygonaceae; Rhei Radix Et Rhizoma(Da-Huang)] | 11.3 |  |  |  |  |
|  | Curcuma Longa L.[Zingiberaceae; Curcumae Longae Rhizoma(Jiang-Huang)] | 11.3 |  |  |  |  |
|  | Carthamus Tinctorius L.[Asteraceae; Carthami Flos (Hong-Hua)] | 9.5 |  |  |  |  |
|  | Angelica dahurica (Hoffm.) Benth. & Hook.f. ex Franch. & Sav.[Apiaceae; Angelicae Dahuricae Radix(Bai-Zhi)] | 11.3 |  |  |  |  |
|  | Atractylodes lancea (Thunb.) DC.[Asteraceae; Atractylodis Rhizoma(Cang-Zhu)] | 4.5 |  |  |  |  |
|  | Magnolia officinalis Rehder & E.H.Wilson[Magnoliaceae; Magnoliae Officinalis Cortex(Hou-Po)] | 4.5 |  |  |  |  |
|  | Citrus × aurantium f. deliciosa (Ten.) M.Hiroe[Rutaceae; Citri Reticulatae Pericarpium(Chen-Pi)] | 4.5 |  |  |  |  |
|  | Arisaema erubescens (Wall.) Schott[Araceae; Arisaematis Rhizoma(Tian-Nan-Xing)] | 4.5 |  |  |  |  |
|  | Glycyrrhiza Glabra L.[Fabaceae; Glycyrrhizae Radix Et Rhizoma (Gan-Cao)] | 4.5 |  |  |  |  |
| Antai Gel Ointment^#^ | Cullen corylifolium (L.) Medik.[Fabaceae; Psoraleae Fructus(Bu-Gu-Zhi)] |  | The drugs are crushed into fine powder, and the auxiliary materials are added to make ointment. | Z20080084  (Guangdong batch number) | Registration Standards for Preparations in Medical Institutions | 24 hours per patch |
|  | Cibotium barometz (L.) J.Sm.[Cyatheaceae; Cibotii Rhizoma(Gou-Ji)] |  |  |  |  |  |
|  | Hansenia weberbaueriana (Fedde ex H.Wolff) Pimenov & Kljuykov[Apiaceae; Notopterygii Rhizoma Et Radix(Qiang-Huo)] |  |  |  |  |  |
|  | Rubus chingii Hu[Rosaceae; Rubi Fructus(Fu-Pen-Zi)] |  |  |  |  |  |
|  | Polygala senega L.[Polygalaceae; Polygalae Radix(Yuan-zhi)] |  |  |  |  |  |
|  | Borneolum Syntheticum (Bing-Pian) |  |  |  |  |  |
| Huoxue Powder^#^ | Angelica Biserrata (R.H.Shan & C.Q.Yuan) C.Q.Yuan & R.H.Shan[Apiaceae; Angelicae Pubescentis Radix (Jiang-Du-Huo)] |  | The drugs are crushed into fine powder and mix well. | Z03140384  (Guangdong batch number) | Registration Standards for Preparations in Medical Institutions | powder into paste, external application |
|  | Fritillaria cirrhosa D.Don[Liliaceae; Fritillariae Cirrhosae Bulbus(Chuan-Bei-Mu)] |  |  |  |  |  |
|  | Magnolia officinalis Rehder & E.H.Wilson[Magnoliaceae; Magnoliae Officinalis Cortex(Hou-Po)] |  |  |  |  |  |
|  | Commiphora Myrrha (T.Nees) Engl.[Olivaceae; Myrrha(Mo-Yao)] |  |  |  |  |  |
|  | Boswellia Sacra Flück. [Burseraceae; Olibanum (Ru-Xiang)] |  |  |  |  |  |
|  | Aconitum carmichaelii Debeaux[Ranunculaceae; Aconiti Radix(Chuan-Wu)] |  |  |  |  |  |
|  | Chaenomeles speciosa (Sweet) Nakai[Rosaceae; Chaenomelis Fructus(Mu-Gua)] |  |  |  |  |  |
| Daiwenjiu Ointment* | Capsicum annuum L.[Solanaceae; Capsici Fructus(La-Jiao)] | 15.4 | Capsicum annuum L.[Solanaceae; Capsici Fructus(La-Jiao)], Cinnamomum verum J.Presl[Lauraceae; Cinnamomi Cortex(Rou-Gui)], and Zingiber officinale Roscoe[Curcumaceae; Zingiberis Rhizoma Recens(Sheng-Jiang)] are crushed into coarse powder respectively, and extracted with ethanol to make a thick paste. Add Cinnamon Oil (Rou-Gui You) to the thick paste and mix well. | Z43020966 | Chinese Pharmacopoeia 2010 Edition Part One | Apply according to acupoints |
|  | Cinnamomum verum J.Presl[Lauraceae; Cinnamomi Cortex(Rou-Gui)] | 3.0 |  |  |  |  |
|  | Zingiber officinale Roscoe[Zingiberaceae; Zingiberis Rhizoma Recens(Sheng-Jiang)] | 81.1 |  |  |  |  |
|  | Cinnamon Oil (Rou-Gui You) | 0.4 |  |  |  |  |

Note: *Drugs' information comes from "China Pharmacopoeia 2020 edition"; ^$^Drugs' information comes from "Guangdong Province Medical Institution Preparation Standard"; ^#^Drugs have not been included in "China Pharmacopoeia" or "Guangdong Medical Institution Preparation Specification", the relevant information of which comes from invention patents. Referring to the publication method of "Chinese Pharmacopoeia", we briefly describe the prescription and preparation method if it involves confidential technology.

Table S2 Fingerprinting methods related to Chinese preparations in this study

| **Medication Type** | **Fingerprinting Methods** |
| --- | --- |
| **Oral Chinese medicine** |  |
| Duhuo Jisheng Mixture* | **High-performance liquid chromatography (HPLC)**: Octadecylsilane bonded silica gel is used as filler; acetonitrile-0.1% phosphoric acid solution (14:86) is used as mobile phase; detection wavelength is 230nm. Paeoniflorin is used as the reference substance. Inject 10 μL each of the test substance and the reference substance solution for detection. Every 1ml of this product contains Paeonia Lactiflora Pall.[Paeoniaceae; Paeoniae Radix Alba (Bai-Shao)], calculated as paeoniflorin (C23H28O11), not less than 0.30mg. |
|  | **Thin-layer chromatography (TC)**: Taking Angelica Biserrata (R.H.Shan & C.Q.Yuan) C.Q.Yuan & R.H.Shan[Apiaceae; Angelicae Pubescentis Radix (Du-Huo)], Angelica sinensis (Oliv.) Diels[Apiaceae; Angelicae Sinensis Radix (Dang-Gui)], Conioselinum Anthriscoides 'Chuanxiong'[Apiaceae; Chuanxiong Rhizome (Chuan-Xiong)], Glycyrrhiza Glabra L.[Fabaceae; Glycyrrhizae Radix Et Rhizoma (Gan-Cao)] and paeoniflorin as controls, in the chromatogram of the test product, fluorescent stripes of the same color appear on the corresponding positions of the chromatogram of the reference product. |
| Yuxuebi Tablet* | **HPLC:** Octadecylsilane bonded silica gel is used as filler; methanol-0.2mol/L ammonium acetate (12:88) (concentrated sulfuric acid to adjust the pH value to 2.2) is used as mobile phase; the detection wavelength is 280nm. Danshensu sodium is used as the reference substance. Inject 10 μL each of the test substance and the reference substance solution for detection. Each tablet of this product contains Salvia Miltiorrhiza Bunge[Labiatae; Salviae Miltiorrhizae Radix Et Rhizoma (Dan-Shen)], calculated as Danshensu sodium (C9H9O5Na), not less than 0.60mg. |
|  | **TC:** Taking Cyathula Officinalis K.C.Kuan[Amaranthaceae; Cyathulae Radix (Chuan-Niu-Xi) ], Conioselinum Anthriscoides 'Chuanxiong'[Apiaceae; Chuanxiong Rhizome (Chuan-Xiong)] and astragaloside as controls, in the chromatogram of the test product, fluorescent stripes of the same color appear at the positions corresponding to the chromatogram of the reference product. |
| Wangbi Tablet* | **HPLC:** Octadecylsilane bonded silica gel is used as filler; methanol-water-glacial acetic acid (55:44:1) is used as mobile phase; detection wavelength is 270nm. Icariin is used as the reference substance. Inject 10 μL each of the test substance and the reference substance solution for detection. Each tablet of this product contains Epimedium sagittatum (Siebold & Zucc.) Maxim.[Berberidaceae; Epimedii Folium(Yin-Yang-Huo)], calculated as icariin (C33H40O15), not less than 0.20mg. |
|  | **TC**: Taking Angelica Biserrata (R.H.Shan & C.Q.Yuan) C.Q.Yuan & R.H.Shan[Apiaceae; Angelicae Pubescentis Radix (Du-Huo)], paeoniflorin and sarsasapogenin as controls, in the chromatogram of the test product, fluorescent stripes of the same color appear at the positions corresponding to the chromatogram of the reference product. |
| Yougui Capsule* | **HPLC:** Octadecylsilane bonded silica gel is used as filler; tetrahydrofuran-methanol-acetonitrile-0.05% phosphoric acid solution (1:4:8:87) is used as mobile phase; the detection wavelength is 236nm. Loganin was used as the reference substance. Inject 10 μL each of the test substance and the reference substance solution for detection, and the content of loganin is determined. |
|  | **TC:** Taking Angelica sinensis (Oliv.) Diels[Apiaceae; Angelicae Sinensis Radix (Dang-Gui)] and cinnamaldehyde as controls, in the chromatogram of the test product, fluorescent stripes of the same color appear at the positions corresponding to the chromatogram of the reference product. |
| Zhongtongan Capsule^#^ | **TC:** Taking Sauromatum giganteum (Engl.) Cusimano & Hett.[Araceae; Typhonii Rhizoma(Zhi-Bai-Fu-Zi)], Angelica dahurica (Hoffm.) Benth. & Hook.f. ex Franch. & Sav.[Apiaceae; Angelicae Dahuricae Radix(Bai-Zhi)], Gastrodia elata Blume[Orchidaceae; Gastrodiae Rhizoma(Tian-Ma)], Saposhnikovia Divaricata (Turcz. Ex Ledeb.) Schischk.[ Apiaceae; Saposhnikoviae Radix (Fang-Feng)], Hansenia weberbaueriana (Fedde ex H.Wolff) Pimenov & Kljuykov[Apiaceae; Notopterygii Rhizoma Et Radix(Qiang-Huo)] and Panax notoginseng (Burkill) F.H.Chen[Araliaceae; Notoginseng Radix Et Rhizoma(San-Qi)] as controls, in the chromatogram of the test product, fluorescent stripes of the same color appear at the positions corresponding to the chromatogram of the reference product. |
| Zhuifeng Tougu Capsule* | **HPLC:** Octadecylsilane bonded silica gel is used as filler; methanol-water (30:70) is used as mobile phase; detection wavelength is 230nm. Paeoniflorin is used as the reference substance. Inject 20 μL each of the test substance and the reference substance solution for detection, and the content of paeoniflorin is determined. |
|  | **TC:** Taking Hansenia weberbaueriana (Fedde ex H.Wolff) Pimenov & Kljuykov[Apiaceae; Notopterygii Rhizoma Et Radix(Qiang-Huo)], Angelica sinensis (Oliv.) Diels[Apiaceae; Angelicae Sinensis Radix (Dang-Gui)], Conioselinum Anthriscoides 'Chuanxiong'[Apiaceae; Chuanxiong Rhizome (Chuan-Xiong)]and ephedrine hydrochloride as controls, in the chromatogram of the test product, fluorescent stripes of the same color appear at the positions corresponding to the chromatogram of the reference product. |
| Hugu Capsule^1^ | **HPLC:** Chromatographic column is Kromasil 100-5C18 (5um, 250mm×4.6mm), the mobile phase is acetonitrile (A)-1% glacial acetic acid (B), the detection wavelength is 320nm, the flow rate is 0.8 ml/min, and the column temperature is 30 °C. Inject 10 μL each of the test substance and the reference substance solution for detection. Ferulic acid, chlorogenic acid, stilbene glycosides, and icariin are used as reference substances to confirm the chemical composition of the chromatographic peaks. |
| Tenghuang Jiangu Tablet^2^ | **HPLC:** The chromatographic column is ECOSIL C18 (250mm×4.6mm×  5μm), the mobile phase is acetonitrile-water (30:70), the column temperature is 35°C, and the flow rate is 1.0 ml/min, the detection wavelength is 270nm. Icariin is used as the reference substance. Inject 10 μL each of the test substance and the reference substance solution for detection, and the content of icariin is determined. |
|  | **TC:** Taking Rehmannia Glutinosa (Gaertn.) Dc.[Orobanchaceae; Rehmanniae Radix Praeparata (Shu-Di-Huang)] and icariin as controls, in the chromatogram of the test product, fluorescent stripes of the same color appear at the positions corresponding to the chromatogram of the reference product. |
| Dahuoluo Capsule^3^ | **Gas Chromatography (GC)**: Identification of Borneolum Syntheticum (Bing-Pian) and Determination of Muscone Content by GC. |
| Xianling Gubao Capsule^4, 5^ | **HPLC:** Chromatographic column is ACQUITY UPLC BEH Shield RP18 (2.1mm×50mm, 1.7um), the mobile phase is acetonitrile-water (each containing 0.1% formic acid by volume), the flow rate is 0.5 ml/min, the column temperature is 30 °C, the detection wavelength is 0-4.6min, 246nm and 4.6-18min, 270nm. By comparing retention time and UV absorption characteristics, combined with peak purity investigation, the main characteristic peaks in the established fingerprints were accurately identified, and belong to the source of traditional Chinese medicine. |
|  | **TC:** Taking Epimedium sagittatum (Siebold & Zucc.) Maxim.[Berberidaceae; Epimedii Folium(Yin-Yang-Huo)], Salvia Miltiorrhiza Bunge[Labiatae; Salviae Miltiorrhizae Radix Et Rhizoma (Dan-Shen)] and Cullen corylifolium (L.) Medik.[Fabaceae; Psoraleae Fructus(Bu-Gu-Zhi)] as controls, in the chromatogram of the test product, fluorescent stripes of the same color appear at the positions corresponding to the chromatogram of the reference product. |
| **External Chinese medicine** |  |
| Jianbu Xiaozhong Zhitong Oil^$^ | **TC:** Taking L-Menthol (Bo-He-Nao) and Syzygium aromaticum (L.) Merr. & L.M.Perry[Myrtaceae; Caryophylli Flos(Ding-Xiang)] as controls, in the chromatogram of the test product, fluorescent stripes of the same color appear at the positions corresponding to the chromatogram of the reference product. |
| Warming Jingjintong Plaster^$^ | **TC:** Taking Cullen corylifolium (L.) Medik.[Fabaceae; Psoraleae Fructus(Bu-Gu-Zhi)], Phellodendron amurense Rupr.[Rutaceae; Phellodendri Amurensis Cortex(Guan-Huang-Bo) and Borneolum Syntheticum (Bing-Pian) as controls, in the chromatogram of the test product, fluorescent stripes of the same color appear at the positions corresponding to the chromatogram of the reference product. |
| Cooling Jingjintong Plaster^6^ | **TC:** Taking Rheum officinale Baill.[Polygonaceae; Rhei Radix Et Rhizoma(Da-Huang)], Cullen corylifolium (L.) Medik.[Fabaceae; Psoraleae Fructus(Bu-Gu-Zhi)], Phellodendron amurense Rupr.[Rutaceae; Phellodendri Amurensis Cortex(Guan-Huang-Bo) and Borneolum Syntheticum (Bing-Pian) as controls, in the chromatogram of the test product, fluorescent stripes of the same color appear at the positions corresponding to the chromatogram of the reference product. |
| Wentong Ointment^#^ | **HPLC:** Chromatographic column is InertsilODS-SPC18 (150mm×4.6mm, 5μm), the mobile phase is acetonitrile-0.02 mol/L sodium dihydrogen phosphate solution (28:72), the detection wavelength is 246nm, the column temperature is 30 °C and the flow rate is 1.0 mL/min. Psoralen and isopsoralen are used as reference substances. Inject 10 μL each of the test substance and the reference substance solution for detection, and the contents of psoralen and isopsoralen are determined. |
| Tianbai Golden Plaster^$^ | **TC:** Taking Rheum officinale Baill.[Polygonaceae; Rhei Radix Et Rhizoma(Da-Huang)], emodin, Phellodendron amurense Rupr.[Rutaceae; Phellodendri Amurensis Cortex(Huang-Bo), berberine hydrochloride, Curcuma Longa L.[Zingiberaceae; Curcumae Longae Rhizoma(Jiang-Huang)] and curcumin as controls, in the chromatogram of the test product, fluorescent stripes of the same color appear at the positions corresponding to the chromatogram of the reference product. |
| Daiwenjiu Ointment* | **HPLC:** Octadecylsilane bonded silica gel is used as filler, acetonitrile-0.1% phosphoric acid solution (45:55) is used as mobile phase, the column temperature is 35°C, and the detection wavelength is 227nm. Capsaicin is used as the reference substance. Inject 10 μL each of the test substance and the reference substance solution for detection. The chromatographic peak with the same retention time as that of the reference substance chromatographic peak should appear in the test product chromatogram. |
|  | **TC:** Taking cinnamaldehyde as controls, in the chromatogram of the test product, fluorescent stripes of the same color appear at the positions corresponding to the chromatogram of the reference product. |

Note: *Drugs' information comes from "China Pharmacopoeia 2020 edition"; ^$^Drugs' information comes from "Guangdong Province Medical Institution Preparation Standard"; ^#^Drugs' information comes from invention patents. For drugs marked with numbers, the relevant information comes from the corresponding references.

References:

Li B.H., Wu J.D., Zhao W.C., Song L.J. (2013). Qualitative Analysis of Composition in Water Extracts from Hugu Capsule by HPLC. Chinese Journal of Information on Traditional Chinese Medicine. 20(08), 69-71. 10.3969/j.issn.1005-5304.2013.08.025.

Wang Y., Zhao Y., Zheng J.F., Ding Y., Li W.L. (2012). Study on quality standard for Tenghuangjiangu tablets. Strait Pharmaceutical Journal. 24(11), 22-25. 10.3969/j.issn.1006-3765.2012.11.008.

You L.H., Yang B.H., Ye M., Hou Y.,Yang Y.N., Zhao X.X. (2014). Simultaneous Determination of Various Components in Dahuoluo Capsule by GC. China Pharmaceuticals. 23(19), 38-40.

Zhu Q.C., Xu D., Wang E., Sun Y.C., Duan L., Li H.X., et al. (2022). Studies on TLC Identification of Xianlinggubao Capsules. Chinese Journal of Ethnomedicine and Ethnopharmacy. 31(14), 53-56. 10.3969/j.issn.1007-8517.2022.14.zgmzmjyyzz202214013.

He L.L., Yao Z.H., Wu X.M., Zhou X.Q., Zhang W., Dai Y., et al. (2016). UPLC-PDA Fingerprint of Xianling Gubao Capsules. Chinese Pharmaceutical Journal. 51(18), 1551-1556.

1. Wang L., Zhou Y., Yuan W.B. (2021). Optimization of Extraction Technique and Identification by Thin Layer Chromatography for Liangxing Jingjintong Patch. Chinese Medicine Modern Distance Education of China. 19(05), 142-145. 10.3969/j.issn.1672-2779.2021.05.057.

Table S3 Comparison of changes in the outcome measures between the two groups ($\bar{x}\pm s$)

|  | WM Group | | | | WM-CM Group | | | |
| --- | --- | --- | --- | --- | --- | --- | --- | --- |
|  | baseline | 2 weeks | 4 weeks | 6 weeks | baseline | 2 weeks | 4 weeks | 6 weeks |
| TS | 37.62 ± 1.702 | 30.31 ± 1.528^*^ | 27.14 ± 1.446^*^ | 24.71 ± 1.38^*^ | 36.23 ± 0.923 | 25.93 ± 0.781^*#^ | 20.21 ± 0.689^*#^ | 16.36 ± 0.62^*#^ |
| PS | 8.62 ± 0.433 | 6.9 ± 0.387^*^ | 5.99 ± 0.361^*^ | 5.39 ± 0.342^*^ | 8.35 ± 0.236 | 6.14 ± 0.202^*^ | 5.05 ± 0.183^*#^ | 4.1 ± 0.165^*#^ |
| SS^a^ | 2.5 ± 0.188 | 2.061 ± 0.171 | 1.816 ± 0.16 | 1.622 ± 0.151 | 2.05 ± 0.094 | 1.349 ± 0.076 | 1.062 ± 0.068 | 0.816 ± 0.059 |
| PFS | 26.5 ± 1.206 | 21.35 ± 1.083^*^ | 19.34 ± 1.03^*^ | 17.7 ± 0.986^*^ | 25.83 ± 0.658 | 18.44 ± 0.556^*#^ | 14.1 ± 0.486^*#^ | 11.45 ± 0.438^*#^ |
| VAS | 5.05 ± 0.196 | 3.91 ± 0.171^*^ | 3.37 ± 0.159^*^ | 2.97 ± 0.149^*^ | 5.23 ± 0.11 | 3.61 ± 0.091^*^ | 2.86 ± 0.081^*#^ | 2.24 ± 0.072^*#^ |
| PCS | 41.98 ± 1.658 | 45.6 ± 1.801^*^ | 48.25 ± 1.906^*^ | 49.09 ± 1.939^*^ | 39.69 ± 0.866 | 48.73 ± 1.063^*^ | 54.18 ± 1.182^*#^ | 59.75 ± 1.304^*#^ |
| MCS | 66.93 ± 1.985 | 70.63 ± 2.095^*^ | 72.08 ± 2.138^*^ | 71.83 ± 2.131^*^ | 64.79 ± 1.062 | 71.03 ± 1.164^*^ | 74.06 ± 1.214^*^ | 78.36 ± 1.284^#*^ |

Note: TS = WOMAC total score; PS = WOMAC pain score; SS= WOMAC stiffness score; PFS= WOMAC physical function score.

^a^ Due to the significant difference between the WOMAC stiffness values of the two groups at baseline, there is no direct comparison of the scores at each time point here; The results of the comparison of changes from baseline between groups can be found in the article.

Values represent Least Squares Mean ± standard deviation (SD) calculated by GLMM; The multiple comparisons are Bonferroni-adjusted.

Compared with this group before treatment, * indicates *P* < 0.05; Compared with the WM group in the same time period, # indicates *P* < 0.05.

Table S4 The scores of SF-36 scale at all measure points ($\bar{x}\pm s$)

|  | WM Group | | | | WM-CM Group | | | |
| --- | --- | --- | --- | --- | --- | --- | --- | --- |
|  | baseline | 2 weeks | 4 weeks | 6 weeks | baseline | 2 weeks | 4 weeks | 6 weeks |
| PF | 50.71 ± 1.897 | 56.79 ± 2.007^*^ | 59.69 ± 2.058^*^ | 60.46 ± 2.071^*^ | 47.4 ± 1.013 | 60.78 ± 1.147^*^ | 66.75 ± 1.202^*#^ | 71.59 ± 1.245^*#^ |
| RP | 68.05 ± 3.581 | 72.73 ± 3.771 | 73.86 ± 3.787 | 73.19 ± 3.753 | 68.18 ± 2.101 | 79.75 ± 2.205^*^ | 83.92 ± 2.212^*#^ | 86.51 ± 2.196^*#^ |
| BP | 45.77 ± 1.332 | 50.08 ± 1.454^*^ | 54.51 ± 1.583^*^ | 55.44 ± 1.614^*^ | 45.28 ± 0.728 | 53.75 ± 0.863^*#^ | 59.79 ± 0.959^*#^ | 64.37 ± 1.033^*#^ |
| GH | 49.07 ± 1.398 | 49.57 ± 1.412 | 50.33 ± 1.434 | 51.09 ± 1.455 | 50.56 ± 0.796 | 53.30 ± 0.839^*#^ | 55.34 ± 0.871^*#^ | 57.15 ± 0.899^*#^ |
| VT^a^ | 70.18 ± 1.615 | 72.10 ± 1.659 | 72.33 ± 1.664 | 71.61 ± 1.648 | 66.35 ± 0.844 | 70.77 ± 0.9 | 72.55 ± 0.922 | 74.29 ± 0.945 |
| SF | 72.37 ± 2.076 | 75.98 ± 2.179^*^ | 78.66 ± 2.256^*^ | 78.82 ± 2.261^*^ | 74.69 ± 1.184 | 80.1 ± 1.269^*^ | 84.96 ± 1.346^*#^ | 87.85 ± 1.392^*#^ |
| RE^b^ | 55.1 ± 4.715 | 62.25 ± 4.603 | 64.63 ± 4.633 | 65.65 ± 4.443 | 50.88 ± 2.664 | 62.31 ± 2.603 | 65.73 ± 2.558 | 76.32 ± 2.298 |
| MH | 70.92 ± 1.682 | 73.19 ± 1.736^*^ | 73.69 ± 1.747^*^ | 72.22 ± 1.713 | 68.6 ± 0.899 | 72.23 ± 0.946^*^ | 74.4 ± 0.975^*^ | 76.02 ± 0.996^*^ |

Note: ^a^ Due to the significant difference between the VT scores of the two groups at baseline, there is no direct comparison of the scores at each time point here；Table S5 shows the results of the comparison of changes from baseline between groups.

^b^ The treatment * time interaction item in GLMM has no statistical significance, so the model cannot be further used for simple effect analysis and multiple comparisons; The values of RE are obtained based on descriptive statistics as mean ± SD; Table S6 shows the results of the main effects analysis.

Except for RE, values represent Least Squares Mean ± SD calculated by GLMM; The multiple comparisons are Bonferroni-adjusted.

Compared with this group before treatment, * indicates *P* < 0.05; Compared with the WM group in the same time period, # indicates *P* < 0.05.

Table S5 Comparison of the changes in VT score from baseline

|  | the changes in VT score from baseline | | *P* |
| --- | --- | --- | --- |
|  | WM Group | WM-CM Group |  |
| 2 weeks | 1.89 ± 8.139 | 4.5 2 ± 10.775 | 0.013 |
| 4 weeks | 2.19 ± 8.795 | 6.29 ± 12.404 | 0.002 |
| 6 weeks | 1.43 ± 11.215 | 8.05 ± 13.224 | ﹤0.001 |

Note: Mann-Whitney U rank sum test was used for comparison between groups, adjusted by the Bonferroni method, with a test level of 0.017.

Table S6 Main effects analysis of RE scores

|  | coefficient | SE | *t* | *P* | 95% CI | |
| --- | --- | --- | --- | --- | --- | --- |
|  |  |  |  |  | lower limit | Upper limit |
| intercept | 4.451 | 0.024 | 183.944 | ﹤0.001 | 4.404 | 4.498 |
| WM-CM Group | 0.054 | 0.026 | 2.046 | 0.041 | 0.002 | 0.106 |
| WM Group | 0^a^ | - | - | - | - | - |
| 6 weeks | 0.058 | 0.012 | 4.684 | ﹤0.001 | 0.034 | 0.083 |
| 4 weeks | 0.039 | 0.012 | 3.335 | ﹤0.001 | 0.016 | 0.062 |
| 2 weeks | 0.031 | 0.010 | 3.159 | 0.002 | 0.012 | 0.050 |
| 0 weeks | 0^a^ | - | - | - | - | - |

Note: ^a^ Take this thing as a reference value. Taking the WM group as reference, the WM-CM group had a higher RE value (*P* = 0.041, the coefficient was positive); taking the baseline RE as reference, the RE values improved significantly after 2 weeks, 4 weeks, and 6 weeks of treatment, showing a trend toward higher and higher values (the coefficients are all positive, and the values are increasing).


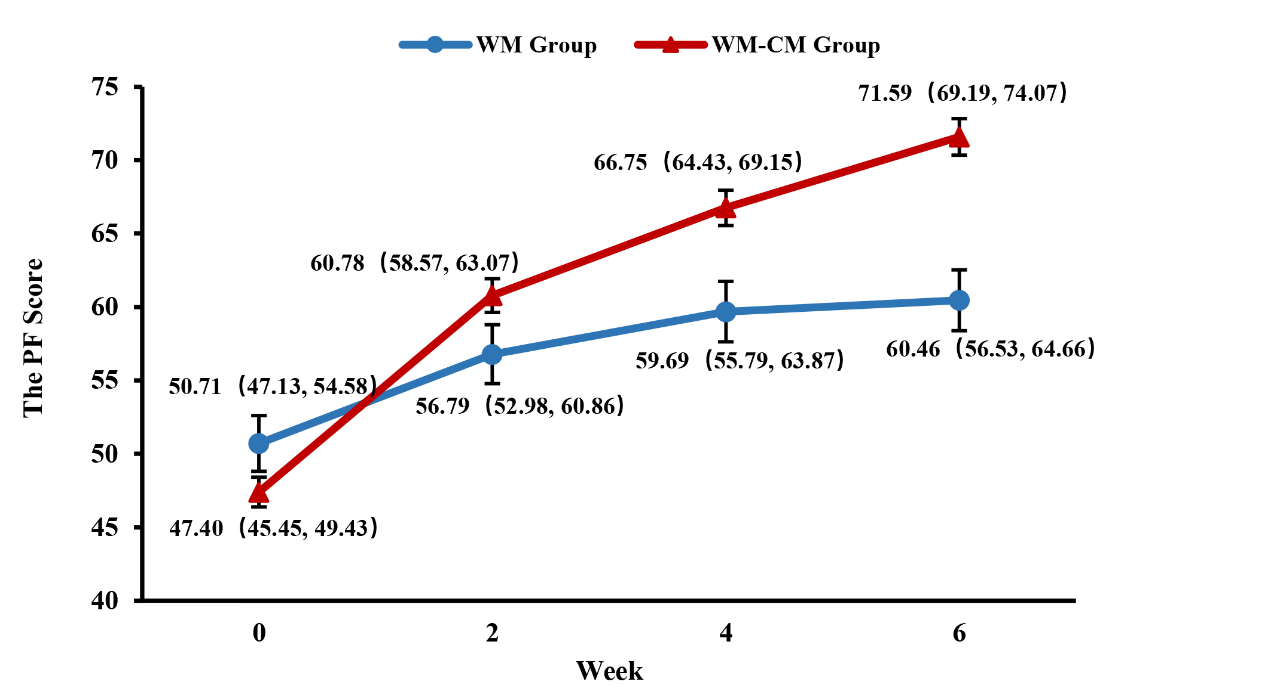


Figure S1. Overall trend of the PF scores

Note: The values shown are least-squares mean calculated based on generalized linear mixed model, with 95% confidence intervals (indicated by error bars) in parentheses.


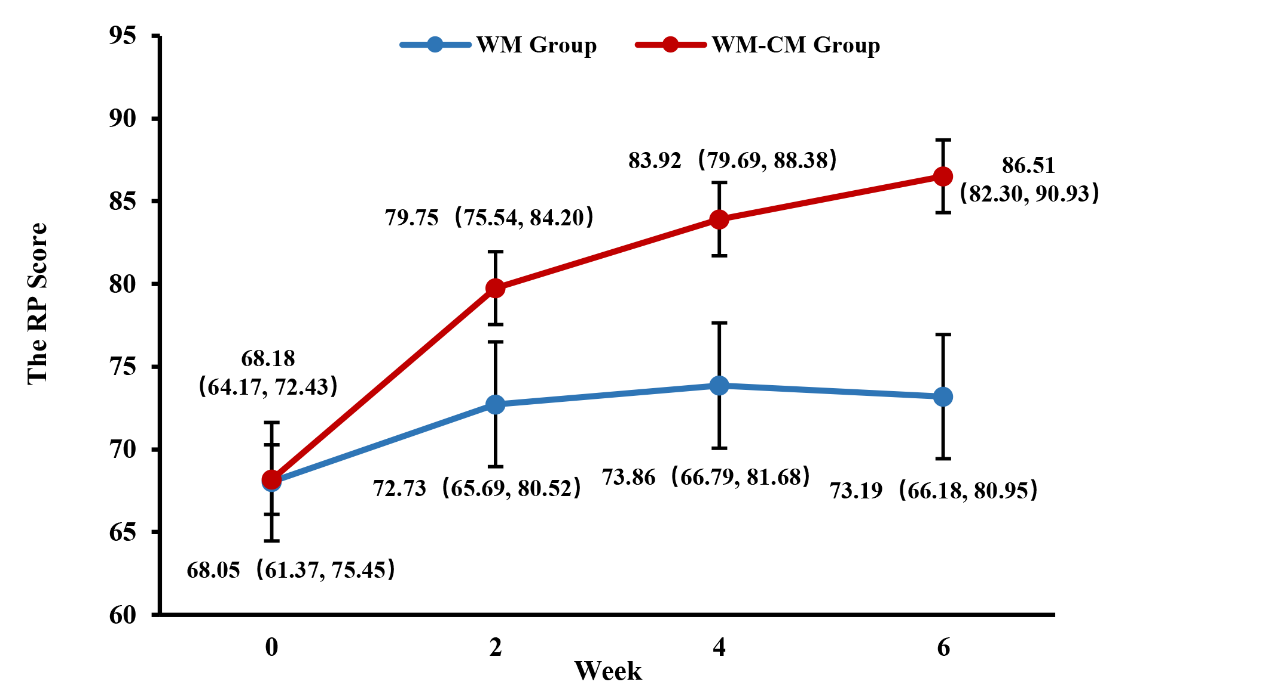


Figure S2. Overall trend of the RP scores

Note: The values shown are least-squares mean calculated based on generalized linear mixed model, with 95% confidence intervals (indicated by error bars) in parentheses.


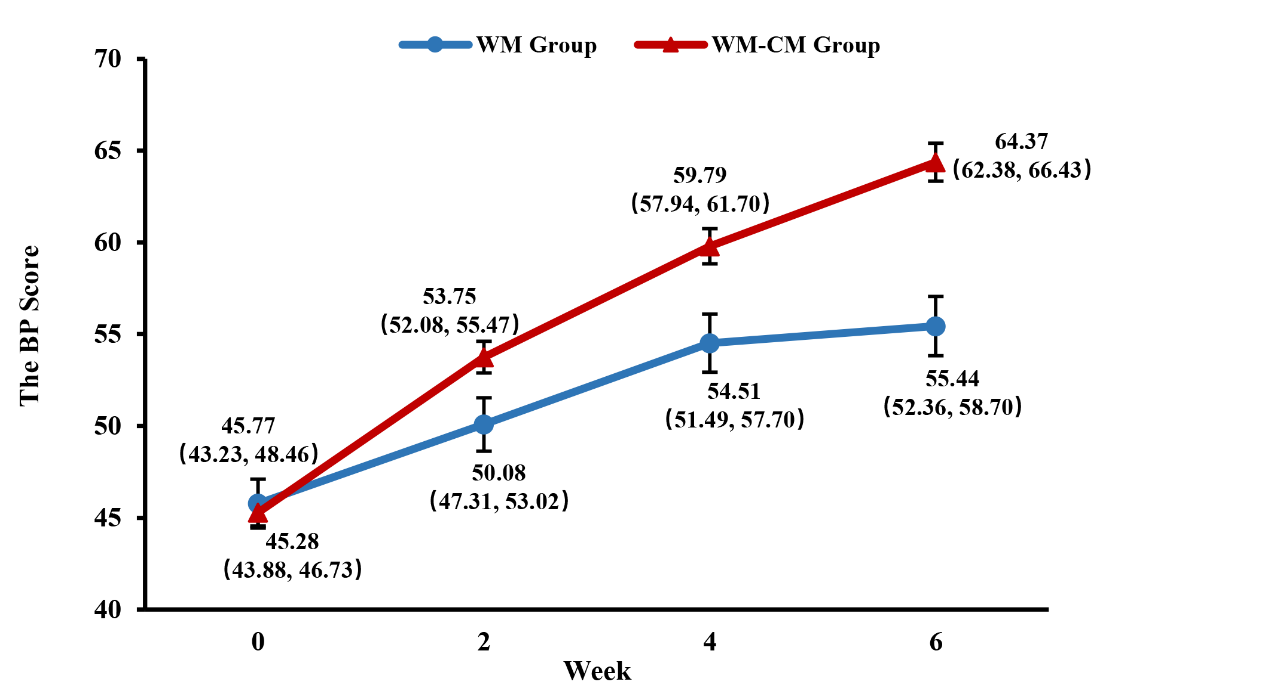
Figure S3. Overall trend of the BP scores

Note: The values shown are least-squares mean calculated based on generalized linear mixed model, with 95% confidence intervals (indicated by error bars) in parentheses.


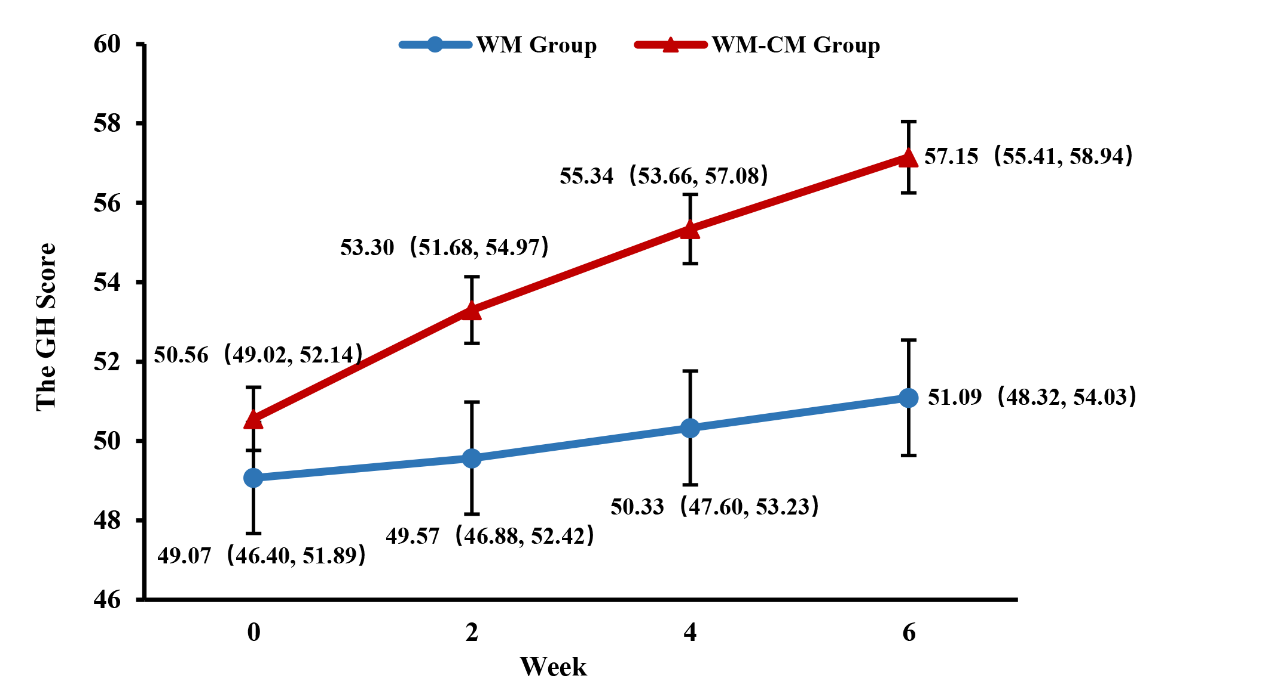


Figure S4. Overall trend of the GH scores

Note: The values shown are least-squares mean calculated based on generalized linear mixed model, with 95% confidence intervals (indicated by error bars) in parentheses.


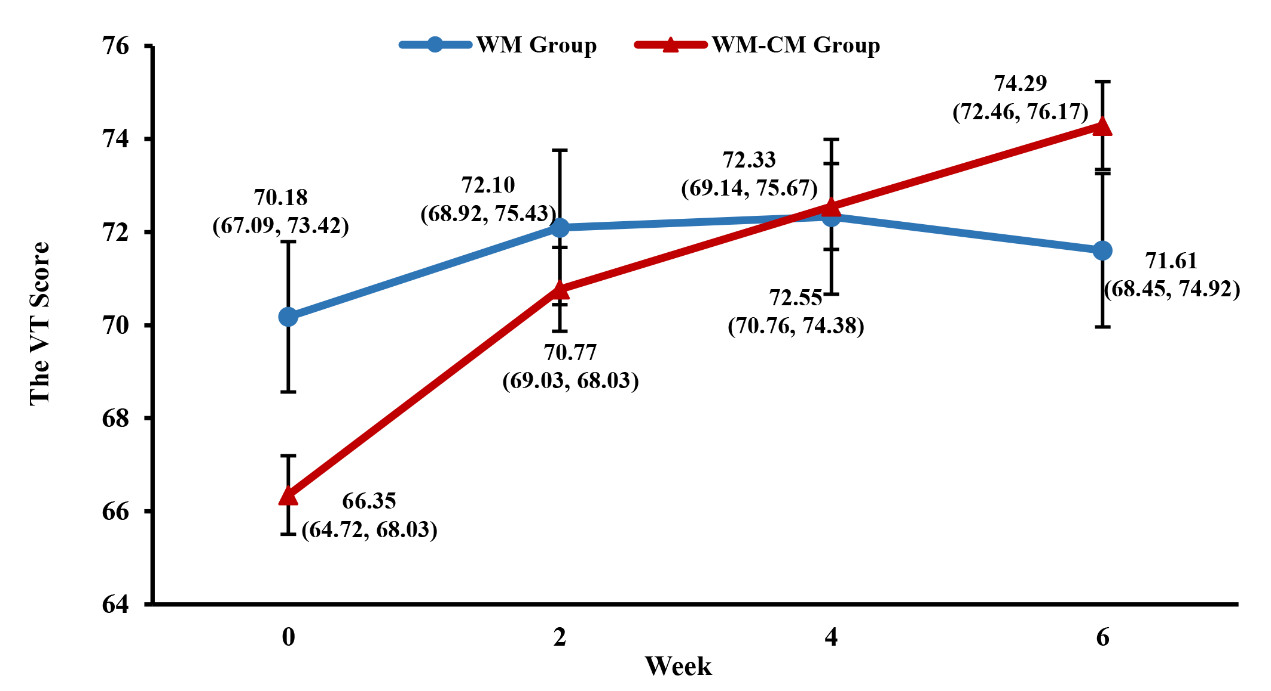


Figure S5. Overall trend of the VT scores

Note: The values shown are least-squares mean calculated based on generalized linear mixed model, with 95% confidence intervals (indicated by error bars) in parentheses.


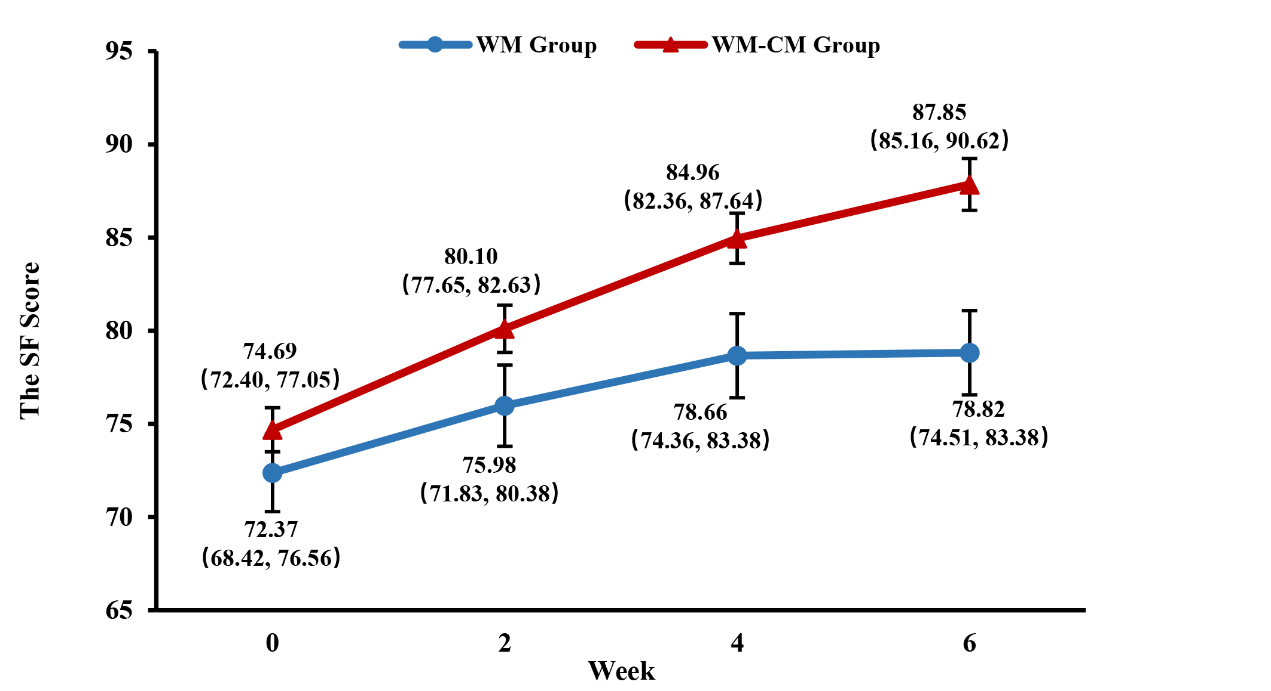


Figure S6. Overall trend of the SF scores

Note: The values shown are least-squares mean calculated based on generalized linear mixed model, with 95% confidence intervals (indicated by error bars) in parentheses.


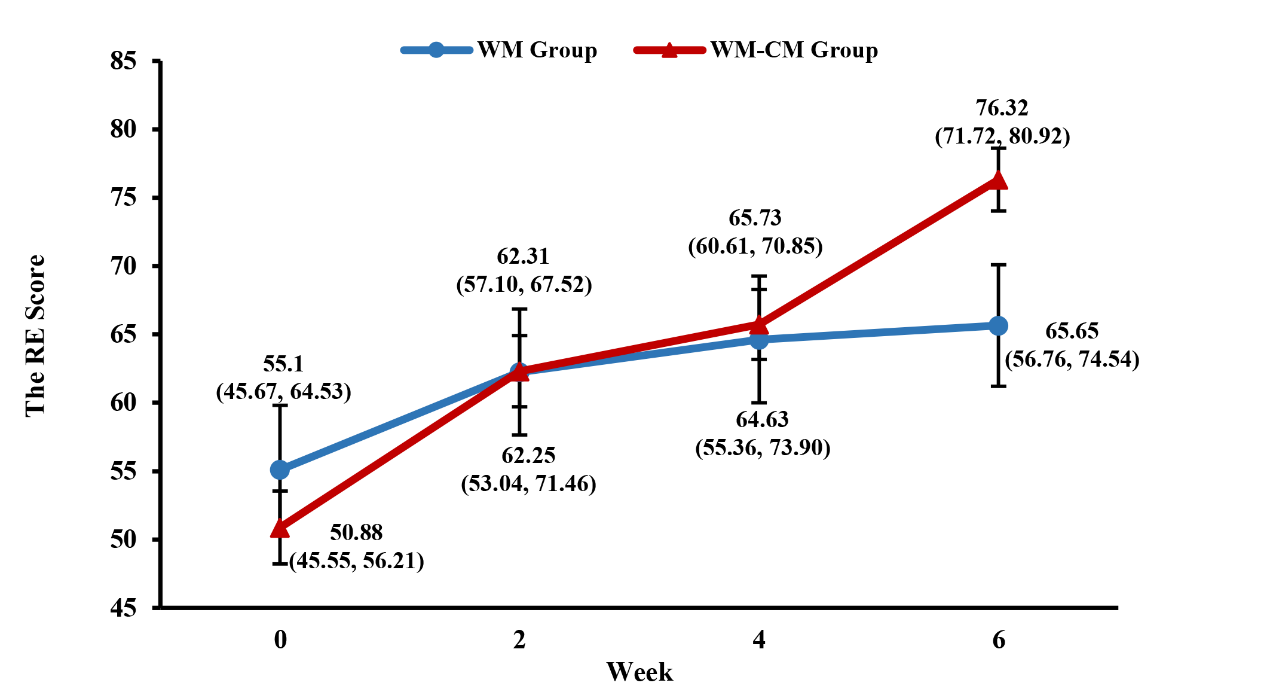


Figure S7. Overall trend of the RE scores

Note: The values are mean obtained based on descriptive statistics, with 95% confidence intervals (indicated by error bars) in parentheses.


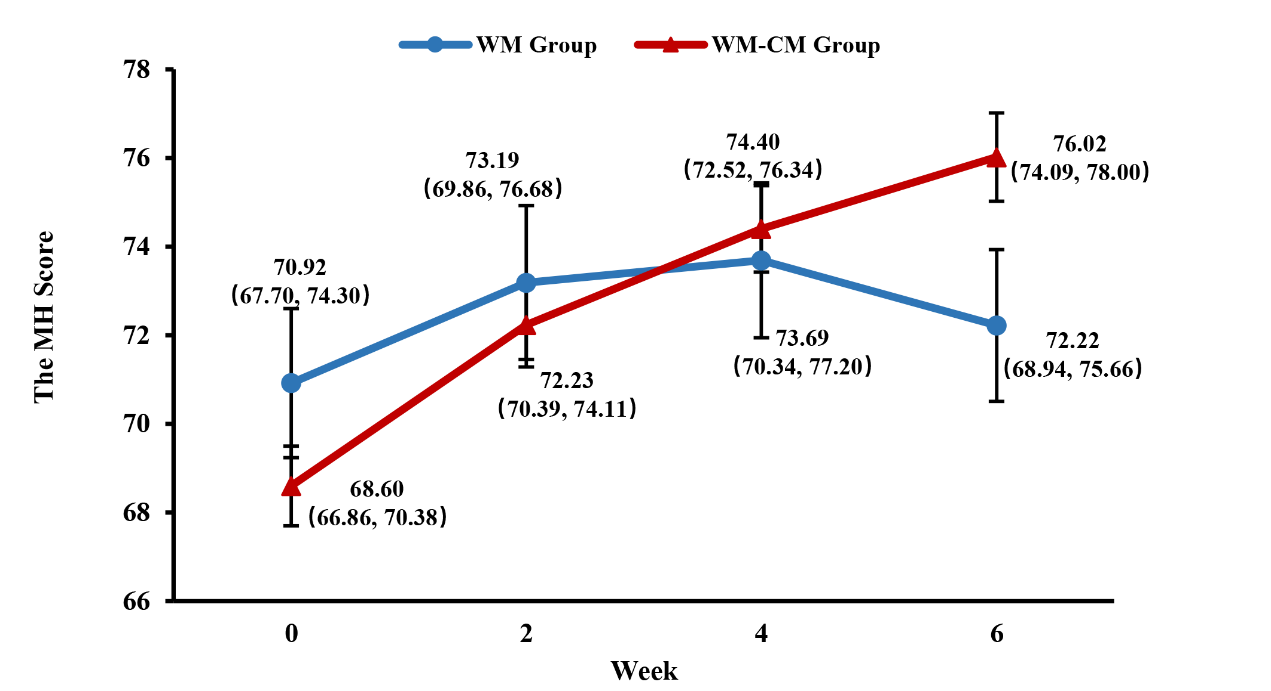


Figure S8. Overall trend of the MH scores

Note: The values shown are least-squares mean calculated based on generalized linear mixed model, with 95% confidence intervals (indicated by error bars) in parentheses.
